# SIRT1 and HSP90α feed-forward circuit safeguards chromosome segregation integrity in diffuse large B cell lymphomas

**DOI:** 10.1038/s41419-023-06186-0

**Published:** 2023-10-11

**Authors:** Emilia Białopiotrowicz-Data, Monika Noyszewska-Kania, Ewa Jabłońska, Tomasz Sewastianik, Dorota Komar, Sonia Dębek, Filip Garbicz, Magdalena Wojtas, Maciej Szydłowski, Anna Polak, Patryk Górniak, Przemysław Juszczyński

**Affiliations:** 1 Department of Experimental Hematology, Institute of Hematology and Transfusion Medicine, Warsaw, Poland; 2 Department of Diagnostic Hematology, Institute of Hematology and Transfusion Medicine, Warsaw, Poland

**Keywords:** B-cell lymphoma, Chromosome segregation

## Abstract

Diffuse large B-cell lymphoma (DLBCL) is the most common aggressive non-Hodgkin lymphoma in adults, exhibiting highly heterogenous clinical behavior and complex molecular background. In addition to the genetic complexity, different DLBCL subsets exhibit phenotypic features independent of the genetic background. For example, a subset of DLBCLs is distinguished by increased oxidative phosphorylation and unique transcriptional features, including overexpression of certain mitochondrial genes and a molecular chaperone, heat shock protein HSP90α (termed “OxPhos” DLBCLs). In this study, we identified a feed-forward pathogenetic circuit linking HSP90α and SIRT1 in OxPhos DLBCLs. The expression of the inducible HSP90α isoform remains under SIRT1-mediated regulation. SIRT1 knockdown or chemical inhibition reduced HSP90α expression in a mechanism involving HSF1 transcription factor, whereas HSP90 inhibition reduced SIRT1 protein stability, indicating that HSP90 chaperones SIRT1. SIRT1-HSP90α interaction in DLBCL cells was confirmed by co-immunoprecipitation and proximity ligation assay (PLA). The number of SIRT1-HSP90α complexes in PLA was significantly higher in OxPhos- dependent than -independent cells. Importantly, SIRT1-HSP90α interactions in OxPhos DLBCLs markedly increased in mitosis, suggesting a specific role of the complex during this cell cycle phase. RNAi-mediated and chemical inhibition of SIRT1 and/or HSP90 significantly increased the number of cells with chromosome segregation errors (multipolar spindle formation, anaphase bridges and lagging chromosomes). Finally, chemical SIRT1 inhibitors induced dose-dependent cytotoxicity in OxPhos-dependent DLBCL cell lines and synergized with the HSP90 inhibitor. Taken together, our findings define a new OxPhos-DLBCL-specific pathogenetic loop involving SIRT1 and HSP90α that regulates chromosome dynamics during mitosis and may be exploited therapeutically.

## Introduction

Diffuse large B-cell lymphoma (DLBCL) is the most common type of non-Hodgkin lymphoma in adults, with striking clinical, transcriptional and genetic heterogeneity [1, 2]. Based on the similarities in tumor cell gene expression profiles to a putative cell of origin (COO), DLBCLs can be classified as either germinal center B-cell-like (GCB) or activated B-cell-like (ABC) DLBCL subtypes [3–6]. More recent studies utilizing multiplatform analyses, combining genomics and transcriptomics revealed additional, previously unrecognized substructure within ABC- and GCB- DLBCLs [7–9]. In addition, DLBCLs exhibit phenotypic features that are independent of their genetic background. Using a gene expression signatures and a set of unbiased clustering approaches (“consensus clustering”), DLBCLs were classified into distinct molecular subtypes that differ in energy source utilization and bioenergetic profiles (comprehensive cluster classification – CCC) [10]. Importantly, the CCC and COO classifications did not overlap, highlighting the fact that they reflect distinct aspects of DLBCL biology. The CCC classification distinguishes tumors that favor oxidative phosphorylation (OxPhos), utilize fatty acids as energy sources, have higher ATP content, and increased thioredoxin and glutathione levels [10–12]. The OxPhos tumors are not addicted to “canonical” survival pathways such as B-cell receptor signaling (BCR); instead, perturbation of energy source supply, inhibition of OxPhos, mitochondrial translation or reactive oxygen utilization pathways was selectively toxic to these tumors, indicating that OxPhos signature is a bona fide survival program activated in these lymphomas [11–13].

One of the key genes of the core signature characterizing OxPhos-dependent tumors is *HSP90AA1*, encoding stress-inducible cytosolic isoform of heat shock protein 90, HSP90α. Inducible HSP90A transcription is controlled by the heat shock factor 1 (HSF1) [14]. Although HSF1 was initially characterized as a regulator of cellular responses to hyperthermia, its activation is also triggered by many other protein-damaging agents, including mitochondria-derived reactive oxygen species and oxidative stress [14]. The molecular chaperone HSP90 is crucial for the stability of many oncogenic proteins, including transcription factors, cell-cycle regulators, BCR pathway components and tyrosine kinases, suggesting that the high expression of HSP90α isoform maintains increased oncogene levels/activities in DLBCL cells [15–17]. In certain cancer cells (including DLBCL), the HSP90 chaperome, i.e ensemble of HSP90-interacting proteins, co-chaperone proteins that assist protein folding in order to ensure their native function, together with “HSP70 chaperome”, assemble into higher-order structures termed “epichaperomes” [18]. By facilitating inducible nucleation and maintenance of functionally-related protein complexes, epichaperomes increase the fitness of the proteome of cancer cells, augment cancer cell metabolism and cellular proliferation, especially under stress [18, 19]. Consistent with this, HSP90 inhibition decreases activity of multiple survival/metabolic pathways, is toxic to a majority of DLBCL cells and synergizes with inhibitors of certain HSP90 client proteins [16, 20, 21].

Sirtuin 1 (silent mating type information regulation 2 homolog, SIRT1) is a NAD(+)-dependent histone deacetylase linking metabolism and survival signaling in cancer [22–24]. Through deacetylation of substrate proteins, SIRT1 controls multiple pathways critical for tumor cell growth, proliferation and survival, including DNA damage, Notch signaling, RNA splicing, transcription, translation, metabolism, cell cycle and chromatin structure [25]. Since multiple SIRT1 substrates are stress-inducible proteins (e.g., p53, NFκB, PGC1α, FOXOs and HSF1), SIRT1 is involved in cellular adaptation to multiple insults, such as starvation, drug exposure or oxidative damage [17, 26–28]. The overexpression of SIRT1 in a subset of DLBCLs was associated with inferior prognosis [29]. However, the mechanisms leading to increased SIRT1 levels in these tumors have not been identified.

In the current study, we identified a feed-forward pathogenetic circuit linking SIRT1 and HSP90α in DLBCLs with OxPhos signature. Given the involvement of SIRT1 and HSP90 in regulation of cellular metabolism, we hypothesized that these proteins might be functionally linked. In line with this hypothesis, we demonstrate that the expression of the inducible HSP90α isoform remains under SIRT1-mediated regulation; in turn, HSP90α chaperones and stabilizes SIRT1, protecting it from ubiquitin-mediated degradation. Surprisingly, we found that the disruption of SIRT1/HSP90α circuit led to improper chromosome segregation during mitosis in OxPhos-DLBCL cells, decreased cell proliferation, and triggered cell death.

## Materials and methods

### Cell lines, cell culture and chemicals

DLBCL cell lines Toledo, Pfeiffer and DHL6 were obtained from American Type Culture Collection (ATCC). Ly1, Ly7 and DHL4 were purchased from Deutsche Sammlung von Mikroorganismen und Zellkulturen. Karpas 422 (K422) was from Sigma-Aldrich and Ly4 was a kind gift from prof. Margaret A. Shipp (Dana-Farber Cancer Institute, Boston, USA). DLBCL cell lines were grown in RPMI-1640 (K422, Toledo, Pfeiffer, DHL4 and DHL6) or Iscove’s Modified Dulbecco’s Medium, (Ly4, Ly1, Ly7). The HEK293T cell line (from ATCC) was maintained in Dulbecco’s Modified Eagle Medium. All cell lines were authenticated by STR profiling and routinely tested for mycoplasma contamination. All culture media were purchased from Lonza and supplemented with heat-inactivated 20% (Ly4) or 10% (the remaining cell lines) fetal bovine serum (FBS), 100 U/mL penicillin, 100 U/mL streptomycin and 25 mmol/L HEPES buffer (all from Lonza). Cell lines were grown in a humidified atmosphere at 37 °C with 5% CO2. SIRT1 activator (SRT-2183) and SIRT1 inhibitors EX-527 (selisistat) and tenovin-6 were purchased from Selleck Chemicals. Cambinol, nocodazole and cycloheximide (CHX) were from Sigma-Aldrich. All chemicals were aliquoted and stored according to the manufacturer’s recommendations.

### Cell treatment

To induce heat shock, DLBCL cells were seeded 0.5 × 10^6^ cells/ml in fresh medium overnight and then incubated in 42 °C for 2 h on a rotating platform. To investigate the effect of SIRT1 inhibition or activation on HSF1 and *HSP90AA1* expression, the cells were incubated with tenovin-6 (4 μM, overnight) or SRT-2183 (1 μM, 24 h), respectively, or with DMSO control (final concentration in the culture medium 0.01%). Thereafter, the cells were exposed to heat shock as described above. For studying SIRT1 protein degradation, DLBCL cell lines were seeded at the density 0.5 × 10^6^ cells/ml in the presence of 10 μg/ml CHX alone or in combination with 2 μM HSP90 inhibitor (17AAG). The cells were collected for western blot analysis every 3 h from the start of the incubation. To investigate the impact of 17AAG on SIRT1 ubiquitination, the cells were grown as above in the presence or absence of a proteasome inhibitor (0.5 μM MG-132) and 2 μM 17AAG for 9 h, and then analyzed in PLA as described below. Control cells were incubated with an adequate concentration of vehicle (DMSO, 0.025%). To enrich the cell culture in mitotic fraction, DLBCL cells were grown in starvation medium (containing 2% FBS) for 24 h, washed with phosphate-buffered saline (PBS) and suspended in the full medium for 6 h. Subsequently, cells were incubated overnight in 50 ng/ml nocodazole, then washed with PBS and grown for the next 8 h in full media.

### Real time quantitative PCR (RQ-PCR)

RNA was isolated using Gene MATRIX Universal RNA/miRNA Purification Kit (EURx) and transcribed to cDNA with Transcriptor Universal cDNA Master (Roche). Primer sequences are given in Table S2. Transcript abundance was measured using iTaq Universal SYBR Green Supermix (Bio-Rad) and LightCycler 480. 18S RNA was used as a housekeeping control gene. Relative transcript abundance was assessed using the 2^-ΔΔCT^ method as previously described [30].

### Immunoblotting

Cells were centrifuged (300 × g, 5 min, 4 °C), washed with PBS and suspended in RIPA buffer supplemented with Protease Inhibitor and PhosSTOP Phosphatase Inhibitor Cocktail Tablets (Roche) according to the manufacturer’s protocol. The protein extracts were quantified using Pierce BCA Protein Assay Kit (ThermoFisher). Total protein extracts (20 μg–40 μg) were mixed with 4× Laemmli sample buffer supplemented with 2-mercaptoethanol and boiled at 95 °C for 5 min. The prepared samples were SDS-PAGE-separated, electrotransferred to PVDF membranes (Millipore), blocked in 5% bovine serum or non-fat milk, and then immunoblotted with primary and appropriate secondary antibodies (Table S4). Signals were detected and quantified as described previously [30]. Densitometry analysis was performed using Image Studio Lite Quantification Software (Licor). The results obtained for the investigated proteins were normalized to glyceraldehyde-3-phosphate dehydrogenase (GAPDH, loading control).

### RNAi-mediated inhibition of gene expression

Short hairpin RNA (shRNA) were designed using GeneScript siRNA Target Finder (https://www.genscript.com/tools/sirna-target-finder) and shRNA Sequence Designer (Clonetech). Oligo sequences are given in Table S1. Designed oligos were synthesized (Sigma-Aldrich), annealed, digested with BamHI and EcoRI, phosphorylated with T4 polynucleotide kinase and cloned into pSIREN-RetroQ as described previously [31]. Obtained vectors were introduced into DLBCL cell lines using retroviral infection [31]. Infected cells were selected with puromycin, and subsequently subcloned by limiting dilution [32]. Individual subclones were assessed for SIRT1 expression by immunoblotting. For chromosome segregation analysis, SIRT1 and HSP90α genes were targeted using SMARTpool Accell siRNA (Horizon Discovery/PerkinElmer), according to the manufacturer’s protocol [33].

### Co-immunoprecipitation

The list of primers used in PCR amplification of desired SIRT1 fragments with FLAG-tag on the carboxy-terminus and the *HSP90AA1* gene with HA-tag on the carboxy-terminus is provided in Table S3. The PCR products were cloned into the pcDNA3.1(+) vector (Invitrogen) using HindIII and BamHI restriction sites for SIRT1 fragments and NotI and BamHI restriction sites for the *HSP90AA1*-HA gene. All obtained constructs were Sanger-sequenced and confirmed to be correct. The vectors were introduced into the HEK293T cells using X-tremeGENE 9 DNA Transfection Reagent (Sigma-Aldrich) according to the manufacturer’s protocol. After 24 h the cells were collected and lysed using Complete Whole Cell Lysis Buffer (Active Motif). Co-immunprecipitation was performed using Universal Magnetic Co-IP Kit (Active Motif). Freshly prepared protein lysates were precleared with magnetic beads and then incubated with appropriate antibody or IgG control at 4 °C on a rotator. After 1 h the magnetic beads slurry was added and the mixture was incubated for the additional 4 h. Thereafter, the beads were washed, resuspended in 2 x Laemmli sample buffer, boiled at 95 °C for 5 min, and lysates were used for immunoblotting.

### Proximity Ligation Assay (PLA)

HEK293T cells grown on glass slides were transfected as described above. After 24 h, the cells were washed with PBS, fixed with 4% paraformaldehyde in PBS for 15 min and permeabilized with 0.5% Triton X-100 in PBS for 15 min. For DLBCL cells, the fixation and permeabilization was performed in 1.5 ml eppendorfs in suspension, then the cells were layered on Superfrost Plus microscope slides (Thermo Scientific) and incubated overnight at 4 °C to attach. Thereafter, HEK293T cells or DLBCL cells were blocked in Duolink Blocking Solution (Sigma-Aldrich). Next, HEK293T cells transfected with HSP90α-HA and SIRT1-FLAG constructs were incubated with primary anti-FLAG and anti-HA antibodies (Table S4). To study the interaction between endogenous SIRT1 and HSP90α in DLBCL cells, representative OxPhos and non-OxPhos lines were stained with anti-SIRT1 and anti-HSP90α antibodies. To analyze the impact of HSP90 inhibitor (17AAG) on SIRT1 ubiquitination, we used mouse anti-SIRT1 antibody in combination with rabbit antibody detecting Lys48-polyubiquitin chains (Supplementary Table S4). All antibody incubations were performed at 4 °C overnight. Proximity ligation assay (PLA) was performed according to the manufacturer’s protocol (Sigma-Aldrich). A red fluorescent signal (λ_ex_ = 594 nm, λ_em_ = 624 nm) was generated only when HSP90α and SIRT1 were in close proximity (<40 nm). Nuclei were stained with Duolink In Situ Mounting Medium containing 4’,6-diamidino-2-phenylindole (DAPI, Sigma-Aldrich) and the cytoskeleton was labeled with phalloidin (ThermoFisher Scientific). Samples were analyzed using Axio Imager.Z2 fluorescent microscope (Zeiss) and the Isis Fluorescence Imaging System (MetaSystems). The total number of foci per nucleus was analyzed using the Image J software (https://imagej.nih.gov/ij/download.html).

### Chromosome segregation analysis

DLBCL cells were enriched in the mitotic fraction as described above. Next, DLBCL cells with siRNA-blocked SIRT1 and/or HSP90α expression were washed with PBS, grown in full medium for 8 h and analyzed using fluorescence microscopy (Axio Imager.Z2). To block SIRT1 and/or HSP90 chemically, parental (unmodified) DLBCL cells were incubated with EX-527 (10 μM) and/or 17AAG (2 μM) inhibitors for 8 h. Cells were then fixed, permeabilized, attached to glass slides and stained for α-pericentrin (centrosome marker), α-tubulin (to visualize the mitotic spindle) and DAPI, and then analyzed for the chromosome segregation errors, such as anaphase bridges, lagging chromosomes, and multipolar spindle formation. The number of cells with missegregated chromosomes was presented as the % of the total number of mitotic cells.

### Proliferation and cytotoxicity

For the measurement of cellular proliferation, cell lines were seeded at concentration 0.2 × 10^6^ cells/ml and counted daily using Trypan Blue exclusion assay. To determine cell viability, DLBCL cells were seeded at density of 15,000 cells/100 μl (K422, Ly4, Pfeiffer, DHL4, Ly1, Ly7) or 45 000 cells/100 μl (Toledo, DHL6) in 96-well plates and treated with different concentrations of SIRT1 and HSP90 inhibitors. Control cells were treated with a vehicle (DMSO). After 72 h of incubation cell viability was estimated using CellTiter 96 AQueous Non-Radioactive Cell Proliferation (MTS) assay (Promega) as described before [34]. The combination index (CI) was calculated using CompuSyn software (http://www.combosyn.com; ComboSyn Inc). CI of 0 < CI ≤ 0.900 indicates synergy, 0.900 < CI ≤ 1.100 indicates additivity, and CI ≥ 1.100 indicates antagonism.

### Bioinformatic and statistical analysis

Gene expression dataset available in the public domain was used to assess HSP90α transcript abundance in lymphoma cells [10]. Data were analyzed and visualized using the MORPHEUS software https://software.broadinstitute.org/morpheus/). Statistical comparisons between variables were performed with GraphPad Prism 6 software (GraphPad Software Inc.). Each experiment was performed at least 3 times in triplicates. No data were excluded from the analyses. Following the Shapiro-Wilk normality test and homogeneity variance *F* test, the statistical significance was calculated using Mann–Whitney test, unpaired *t* test or paired *t* test, as indicated in the figures legends. *P* value < 0.05 was considered statistically significant. *p < 0.05, **p < 0.01, ***p < 0.001 and ****p < 0.0001.

## Results

### Expression of HSP90α correlates with the SIRT1 protein level

**Fig. 1:**
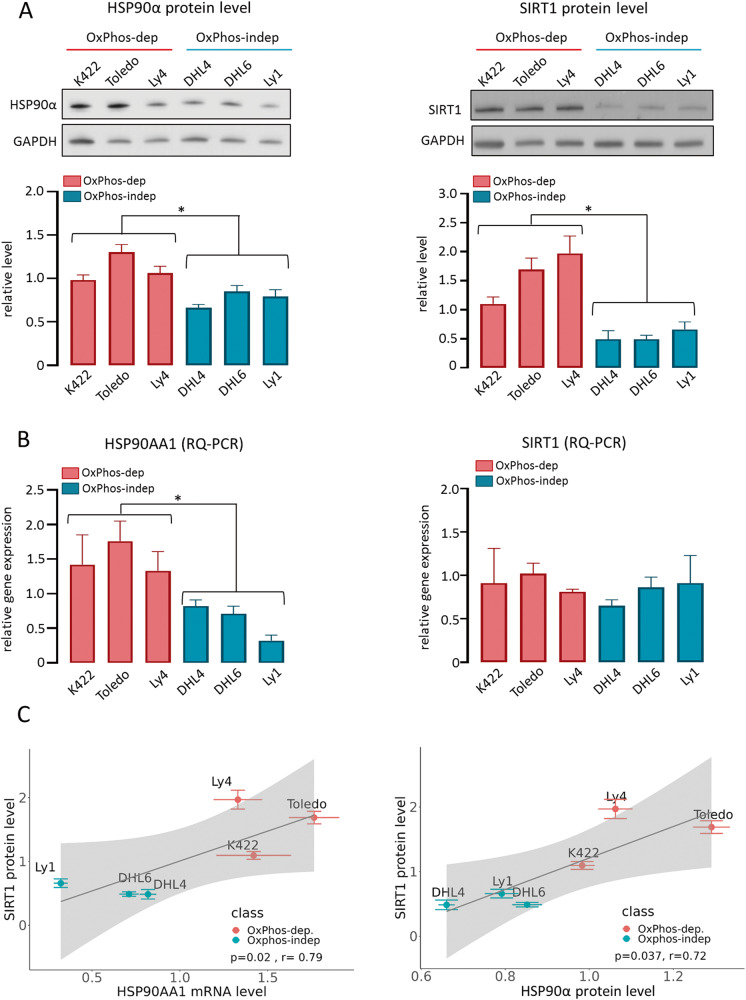
HSP90α transcript and protein abundance correlate with the SIRT1 protein level in DLBCL cell lines. **A** HSP90α (left) and SIRT1 (right) protein levels in a panel of OxPhos- dependent (OxPhos-dep) and -independent (OxPhos-indep) DLBCL cell lines. Upper panels show representative immunoblots from three independent experiments; GAPDH was used as a loading control. Lower panels represent relative quantifications of band intensities from digital images. Relative HSP90α and SIRT1 protein expression was calculated as a ratio of HSP90α or SIRT1 band intensity to GAPDH band intensity. The individual values were normalized to the averaged value obtained for all analyzed DLBCL cell lines, which was assigned as arbitrary value 1. **B** Transcript abundance for HSP90α (left) and SIRT1 (right) in OxPhos- dependent and -independent DLBCL cell lines. Relative abundance of HSP90α and SIRT1 transcripts was determined using 2^−ΔΔCT^ method, with 18S ribosomal RNA used as a reference gene. The individual values were normalized to the average obtained from all investigated cell lines, which was assigned as value 1. In (**A**) and (**B**) bars represent averages from 3 independent experiments ± standard deviation (SD). Statistical comparisons were performed using unpaired *t* test, * indicates *p* < 0.05. **C** The correlation between HSP90α transcript (left) and protein level (right) with the SIRT1 protein abundance; r - the Spearman correlation coefficient.

To understand the mechanism of HSP90α overexpression in a subset of DLBCLs, we first evaluated the HSP90AA1 transcript and protein level in a panel of OxPhos -dependent (K422, Toledo, Ly4, Pfeiffer) or -independent (DHL4, DHL6, Ly1, Ly7) DLBCL cell lines [11, 35]. Similarly to the higher expression of HSP90AA1 in primary OxPhos-reliant cells, three out of four OxPhos-DLBCL cell lines (K422, Toledo, Ly4) demonstrated significantly increased HSP90AA1 transcript and HSP90α protein levels (relative values greater than the average for all cell lines), whereas among the 4 OxPhos-independent cell lines DHL4, DHL6 and Ly1 showed lower HSP90α gene and protein expression (relative values lower than the average for all cell lines, Supplementary Fig. S1). We therefore chose these six cell lines as representative for OxPhos and non-OxPhos tumors for further investigations (Fig. 1A, B). Since the HSP90AA1 expression is controlled by the HSF1 transcription factor, which is regulated by sirtuin 1 (SIRT1) deacetylase [14, 36], we evaluated gene and protein expression of SIRT1 in these DLBCL cell lines. OxPhos-dependent cell lines exhibited increased levels of SIRT1 protein (Fig. 1A, right). However, in contrast to the SIRT1 protein expression, SIRT1 transcript abundance was similar in all analyzed cell lines, suggesting posttranslational regulation of SIRT1 level in DLBCL (Fig. 1B, right). HSP90AA1 transcript and protein levels correlated with SIRT1 protein level (*p* = 0.02, *r* = 0.79, and *p* = 0.037, *r* = 0.72, respectively, Fig. 1C). This relationship was HSP90α-specific, since neither the transcript nor the protein abundance of the gene encoding the second cytosolic HSP90 isoform HSP90β correlated with the SIRT1 protein level (Supplementary Fig. S2).

### SIRT1 regulates the HSP90AA1 gene expression in OxPhos-DLBCL cells

**Fig. 2:**
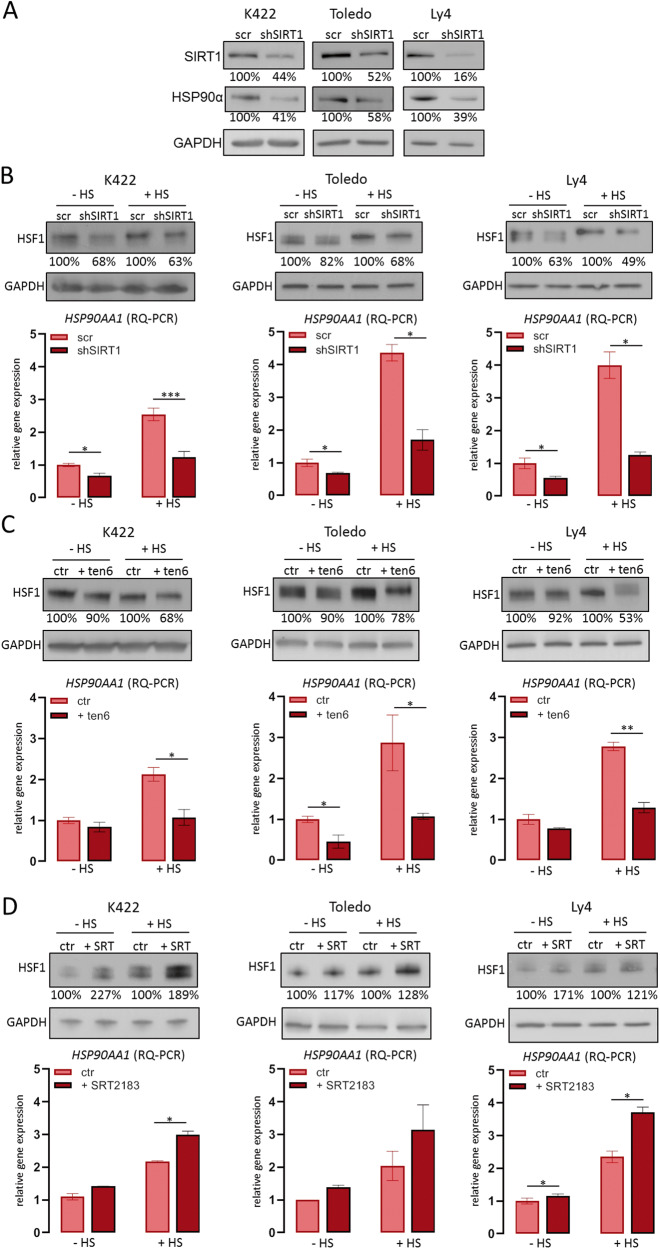
SIRT1 inhibition reduces the HSF1 protein level and decreases the *HSP90AA1* gene expression. **A** SIRT1 knockdown decreases the HSP90α protein level in OxPhos-dependent DLBCL cell lines. **B** SIRT1 knockdown decreases the HSF1 protein level and impairs the HSP90AA1 gene expression in cells in normal conditions (-HS) and after heat shock (+HS, 42 °C, 2 h). **C** Inhibition of SIRT1 with tenovin-6 (ten6) reduces the HSF1 protein level and impairs expression of the *HSP90AA1* gene in cells in normal conditions (-HS) and after heat shock (+HS). Cells were incubated with 4 µM tenovin 6 (ten6) or vehicle (DMSO) overnight and then lysed. Heat shock was induced by 2 h incubation at 42 °C. Protein extracts and RNA were used for western blots and RQ-PCR analyses. **D** SIRT1 activator, SRT-2183, increases HSF1 protein level and *HSP90AA1* gene expression in OxPhos-dependent DLBCL cell lines. Cells were incubated with 1 µM SRT-2183 or DMSO for 24 h. Heat shock was induced as described above. In **A**, **B**, **C** and **D**, representative immunoblots from 3 independent experiments are shown. GAPDH was used as a loading control. Values beneath the blots indicate densitometric bands intensities, in which HSF1 level in controls (scr or ctr) was assigned the value 100%. RQ-PCR graphs in **B**, **C** and **D** represent an average of three independent experiments ± SDs. **** for *p* < 0.0001, ** for *p* < 0.01 and * for *p* < 0.05. The statistical comparisons were performed with *t* test.

SIRT1-mediated deacetylation of HSF1 transcription factor increases HSF1 stability and favors the expression of multiple heat shock proteins [36, 37]. These earlier studies and the correlation between the HSP90AA1 and the SIRT1 protein level in DLBCL cells led us to hypothesize that HSP90α and SIRT1 proteins might be linked in a common regulatory circuit. Consistent with this hypothesis, SIRT1 knockdown markedly decreased HSP90α protein level (Fig. 2A). To understand the mechanism linking SIRT1 with decreased HSP90α levels, we evaluated HSF1 expression after SIRT1 knockdown. SIRT1 blockade in either unstressed cells or in cells exposed to heat shock stress markedly decreased HSF1 levels (Fig. 2B). SIRT1-knockdown cells exhibited decreased HSP90AA1 mRNA levels, particularly following heat shock (Fig. 2B). Incubation of unstressed or heat-shock-exposed OxPhos-DLBCL cell lines with sirtuin inhibitor, tenovin-6 (ten6) similarly decreased the HSF1 protein level and reduced the HSP90AA1 transcript abundance (Fig. 2C). Next, we asked whether increasing SIRT1 activity would cause the opposite effects to those observed for SIRT1 knockdown or chemical inhibition. As shown in Fig. 2D, incubation of OxPhos-dependent DLBCL cell lines with SIRT1 activator (SRT-2183) increased HSF1 protein level and induced HSP90α gene expression. Heat shock further augmented these differences (Fig. 2D). Taken together, these results indicate that increased SIRT1 protein level is at least in part responsible for the *HSP90AA1* gene overexpression in OxPhos-DLBCLs.

### HSP90α chaperones SIRT1

**Fig. 3:**
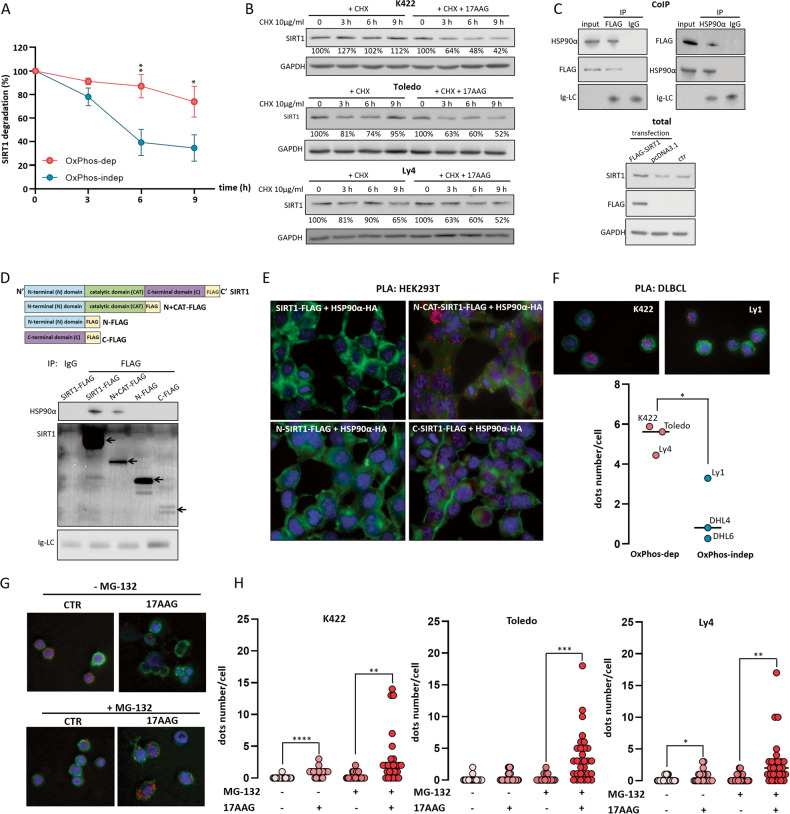
HSP90α interacts with SIRT1 protein and increases its stability. **A** Increased SIRT1 protein stability in OxPhos-DLBCL cell lines (K422, Toledo, Ly4) compared to OxPhos-independent cells (DHL4, DHL6, Ly1). Cells were grown in the presence of 10 µg/ml cycloheximide (CHX) and collected for western blot analysis every 3 h from the start of the incubation. Kinetics of SIRT1 degradation was assessed in each cell line by immunoblotting and quantified using image densitometry (please refer also to Supplementary Fig. S3). SIRT1 protein expression was assessed relatively to GAPDH and referred to the SIRT1 protein level at 0 h, which was assigned the value of 100%. **B** HSP90 inhibition with 17AAG accelerates SIRT1 protein degradation in OxPhos-dependent DLBCL cell lines. Representative western blots from 3 independent experiments were shown. The relative SIRT1 protein level was assessed using band densitometry as in (**A**). **C** SIRT1 co-immunoprecipitates with HSP90α. Upper: cell lysates from HEK293T cells overexpressing FLAG-tagged SIRT1 were used for co-immunoprecipitation experiments with α-FLAG (left) or α-HSP90α (right) antibodies. The level of light chain immunoglobulin (Ig-LC) was used as a loading control. Input represents 5% of total cell lysate. **D** SIRT1 catalytic domain is responsible for the interaction with HSP90α. Upper: The schematic of designed SIRT1 fragments used for co-immunoprecipitation experiments. Lower: Cell lysates from HEK293T cells overexpressing SIRT1 fragments were used for immunoprecipitation with α-FLAG or IgG (negative control) and immunoblotted with α-HSP90α and α-SIRT1 antibodies. Arrows indicate SIRT1 fragments. **E** SIRT1-HSP90α protein complexes in HEK293T cells identified using proximity ligation assay (PLA). HEK293 cells were transduced with vectors carrying human influenza hemagglutinin tagged HSP90α (HSP90α-HA) and either the full length SIRT1-FLAG, or the SIRT1 fragment comprising the N-terminal domain, or both the N-terminal and the catalytic domains (N-CAT SIRT1-FLAG). The SIRT1-HSP90α complexes are shown in red, the nuclei are stained blue (DAPI) and the actin filaments are shown in green (Alexa Fluor 488 - phalloidin). Original magnification was 50×. Representative images of three independent experiments are shown. Please, see also associated Supplementary Fig. S4A. **F** The number of SIRT1-HSP90α complexes is higher in OxPhos- dependent than -independent DLBCL cell lines. Upper: representative images with SIRT1-HSP90α PLA complexes in Oxphos-dependent (K422) or -independent (Ly1) cell lines. Original magnification was 50×. Lower: the summary graph of three independent experiments showing the higher number of SIRT1-HSP90α complexes in OxPhos-dependent cell lines compared to OxPhos-independent cells. Vertical line indicates average number of PLA complexes. Thirty cells were analyzed for each cell line. Statistical comparison was performed using *t* test; * for *p* < 0.05 (**G**) SIRT1 ubiquitination increases after HSP90 inhibition in OxPhos-dependent cells. K422 cells were seeded 0.5 × 10^6^ cells/ml and treated with HSP90 inhibitor (0.5 µM 17AAG) in the presence/absence of proteasome inhibitor (0.5 µM MG-132) for 9 h. SIRT1 ubiquitination was assessed by proximity ligation assay. Original magnification was 50×. **H** Summary plots showing increased SIRT1 ubiquitination after incubation with 17AAG in K422, Toledo and Ly4. Representative graphs of three independent experiments were shown. For each condition, 30 cells were analyzed. Statistical comparisons were performed using *t* test; *** for *p* < 0.001, ** for *p* < 0.01 and * for *p* < 0.05.

Lack of differences in SIRT1 transcript abundance between OxPhos-dependent and -independent cell lines (Fig. 1B, right panel) suggested that SIRT1 protein overexpression may result from increased protein stability. To test this hypothesis, we incubated DLBCL cells with a protein biosynthesis inhibitor, CHX and evaluated time-course changes in SIRT1 protein abundance using immunoblotting. OxPhos-DLBCL cell lines (K422, Ly4, Toledo) showed markedly increased SIRT1 stability when compared to OxPhos-independent DLBCL cell lines with lower HSP90α level (DHL4, DHL6, Ly1; Fig. 3A and Supplementary Fig. S3). Given the HSP90α molecular chaperone function, we hypothesized that HSP90α protein might physically interact and stabilize SIRT1 in OxPhos-DLBCLs. We first compared the stability of SIRT1 protein over time in cells incubated with CHX alone or simultaneously treated with the HSP90 inhibitor, 17-N-allyamino-17-demethoxygeldanamycin (17AAG, Fig. 3B). When compared to the CHX-only treated cells, inhibition of HSP90 significantly accelerated SIRT1 protein degradation (Fig. 3B), suggesting that SIRT1 is an HSP90α client. To further investigate HSP90α-SIRT1 interactions, we overexpressed FLAG-tagged SIRT1 in HEK293T cells and pulled down SIRT1-interacting proteins using α-FLAG antibody and HSP90α-interacting proteins using HSP90α antibody. In these experiments, HSP90α co-immunoprecipitated with SIRT1 (Fig. 3C). To specifically identify the SIRT1 fragment responsible for HSP90α interactions, we developed plasmids encoding FLAG-tagged N-terminal, catalytic and C-terminal SIRT1 domains (Fig. 3D). Since the construct encoding the catalytic domain did not produce a stable peptide, we designed an additional plasmid encoding a longer SIRT1 fragment, which comprised N-terminal and catalytic domains (N + CAT-FLAG), and obtained a stable protein product (Fig. 3D). After transduction of HEK293T cells with HSP90α-HA and FLAG-tagged SIRT1 fragment- coding plasmids, we immunoprecipitated SIRT1 fragments using α-FLAG antibody and assessed their interactions with HA-tagged HSP90α. As shown in Fig. 3D, the interaction with HSP90α was observed only for the longest SIRT1 fragment covering the N-terminal and the catalytic domains (N + CAT-FLAG). Given the fact that the isolated N-terminal domain was not able to bind the HSP90α, these results indicate that the SIRT1 catalytic domain is responsible for the interaction with HSP90α (Fig. 3D). To further confirm these results, we performed proximity ligation assay (PLA) in HEK293T cells overexpressing HSP90α-HA and FLAG-tagged SIRT1 (full-length or fragments). Consistent with the co-immunoprecipitation experiments, the SIRT1-HSP90α complexes were detected in cells overexpressing either the full-length SIRT1 protein or N + CAT SIRT1 fragment (Fig. 3E, Supplementary Fig. S4A). Importantly, SIRT1 and HSP90α formed complexes also in lymphoma cells and the number of SIRT1-HSP90α complexes was significantly higher in OxPhos- dependent than -independent DLBCL cell lines (Fig. 3F, Supplementary Fig. S4B).

HSP90α increases the stability of its client proteins by maintaining their proper folding and protecting them from ubiquitination and subsequent proteasomal degradation. To confirm this mechanism in the case of SIRT1, we evaluated the level of ubiquitinated SIRT1 in OxPhos-dependent DLBCL cell lines treated with HSP90 inhibitor 17AAG in the presence/absence of proteasome inhibitor MG-132 using PLA. Inhibition of HSP90 dramatically increased the number of PLA signals, indicating increased SIRT1 ubiquitination (Fig. 3G, H). SIRT1 ubiquitination was further augmented by the proteasome inhibitor (Fig. 3G, H).

### HSP90α and SIRT1 are required for proper chromosome segregation during mitosis

**Fig. 4:**
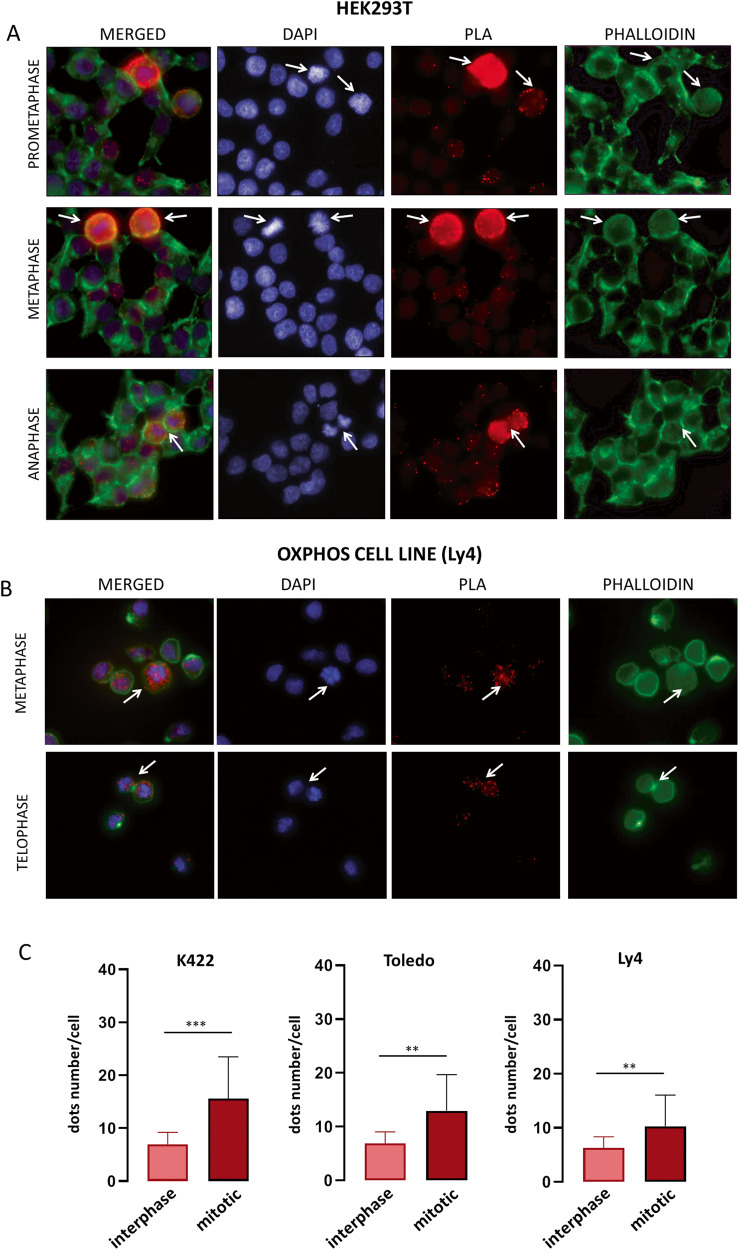
HSP90α-SIRT1 interactions are augmented during mitosis. **A** Increased interaction between SIRT1 and HSP90α in mitotic cells compared to interphase HEK293T cells. HEK293T cells were transfected with SIRT1-FLAG and HSP90α-HA constructs and after 24 h used in PLA experiment to mark HSP90α-SIRT1 complexes with FLAG and anti-HA antibodies. **B** Increased number of HSP90α-SIRT1 complexes in mitotic DLBCL cells. In **A**, **B** and **C**, mitotic cells are marked with white arrows. Original magnification for all images was 50×. **C** Summary plots with statistical comparisons showing significant increase of HSP90α-SIRT1 complexes in mitotic, compared to interphase, OxPhos-dependent cells. Graphs represent three independent experiments. 30 cells were analyzed in each condition. Statistical comparisons were performed using *t* test; *** for *p* < 0.001 and ** for *p* < 0.01.

**Fig. 5:**
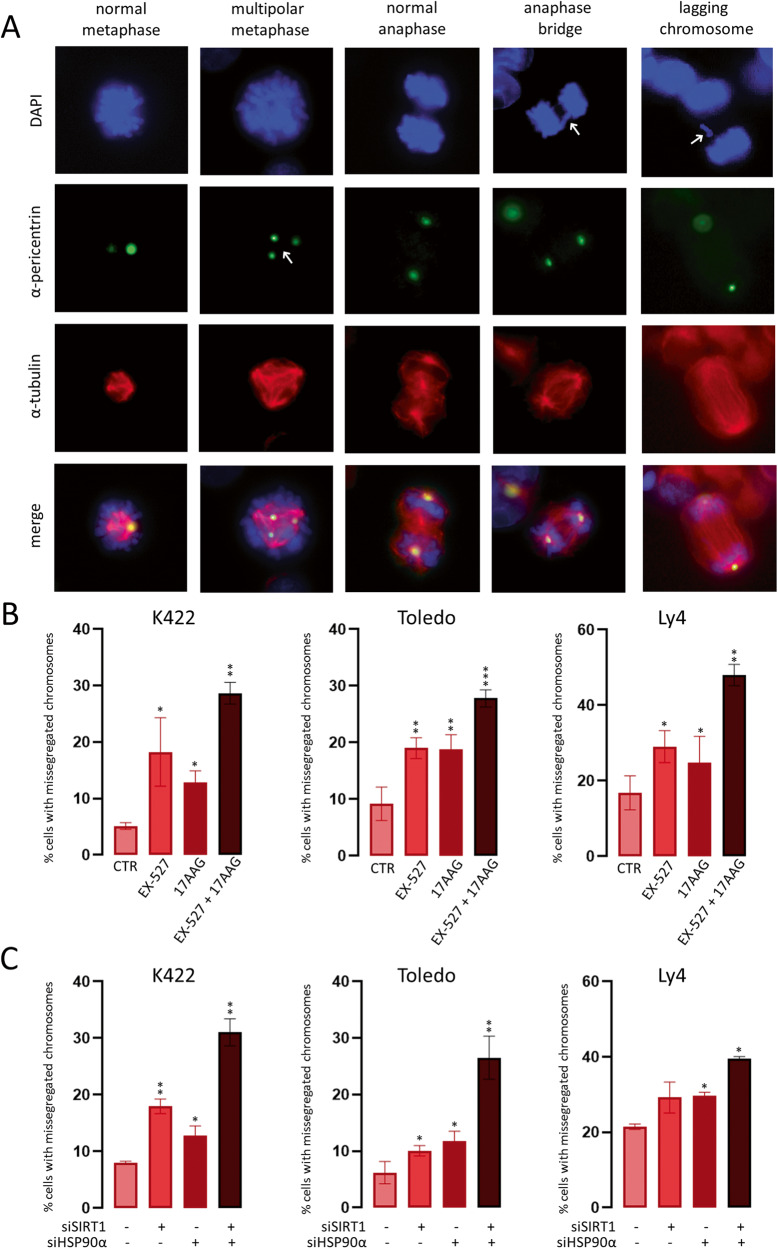
Chromosome segregation errors in cells with blocked SIRT1 and/or HSP90α. **A** The most common chromosome segregation abnormalities in DLBCL cells with inhibited SIRT1 activity. Original magnification was 100×. **B** Summary statistics presenting increase in the fraction of mitotic cells with aberrant chromosome segregation after chemical inhibition of SIRT1 (10 µM EX-527) and/or HSP90 (2 µM 17AAG) in OxPhos-dependent DLBCL cell lines. **C** SiRNA-mediated inhibition of SIRT1 and/or HSP90α significantly increases the number of cells with chromosome segregation errors in OxPhos-DLBCL cell lines K422, Toledo and Ly4. In **B** and **C**, bars represent averages ± SDs from 3 independent experiments. For each condition, 50 mitotic cells in a single experiment were analyzed. The statistical comparisons were performed using *t* test; *** for *p* < 0.001 and ** for *p* < 0.01.

Studying the PLA images, we noticed that mitotic HEK293T cells overexpressing SIRT1 and HSP90α exhibited dramatically increased number of HSP90α-SIRT1 complexes compared to the interphase cells (Fig. 4A). In fact, the number of PLA signals in mitotic HEK cells was so high that it could not be reliably enumerated with the image analysis software (Fig. 4A). The number of HSP90α-SIRT1 complexes was also higher in mitotic than interphase DLBCL cells (Fig. 4B). Importantly, the increase in the number of HSP90α-SIRT1 complexes during mitosis was higher in OxPhos-dependent (*p* < 0.05) than OxPhos-independent cells (Fig. 4B, C, Supplementary Fig. S5B–C). These observations strongly suggested a mitosis-specific function of the SIRT1-HSP90α complex. Of note, SIRT1 regulates the polymerization of microtubules, which is required for proper chromosome segregation during the cell division [38]. Thus, we hypothesized that these proteins may together play a role in ensuring proper chromosome segregation. We thus compared the integrity of mitotic chromosome separation in DLBCL cells in the presence and absence of SIRT1 and/or HSP90α activity. To enrich the cell culture with the mitotic cell fraction, the cells were first synchronized using a starvation medium containing 2% FBS/nocodazole and then treated with 10 µM EX-527 and/or 2 µM 17-AAG for 8 h. Inhibition of SIRT1 significantly increased the fraction of cells with chromosomal segregation errors (Fig. 5). The most common mitotic aberrations included multipolar spindle formation, anaphase bridges and lagging chromosomes (Fig. 5A), which constituted respectively 50%, 26% and 20% of all abnormal mitotic incidents. HSP90 inhibitor, 17AAG, also increased the percentage of cells with defective chromosome separation (Fig. 5B). Combination of EX-527 and 17AAG significantly increased the number of mitotic cells with chromosomal separation errors to 28.37–48% when compared to the effects observed for each of the inhibitors alone (Fig. 5B). We next confirmed these results in DLBCL cells with genetic knockdown of the SIRT1 or/and HSP90α expression (Supplementary Fig. S6). Similarly to the SIRT1 and HSP90α inhibitors, genetic disruption of either of the genes increased the number of cells with chromosomes separation errors (Fig. 5C). Simultaneous silencing of both genes further increased the percentage of mitotic aberrations in OxPhos-DLBCL cells (Fig. 5C, Supplementary Fig. S6).

### Combinatorial inhibition of SIRT1 and HSP90 induces synergistic toxicity in OxPhos-DLBCLs

**Fig. 6:**
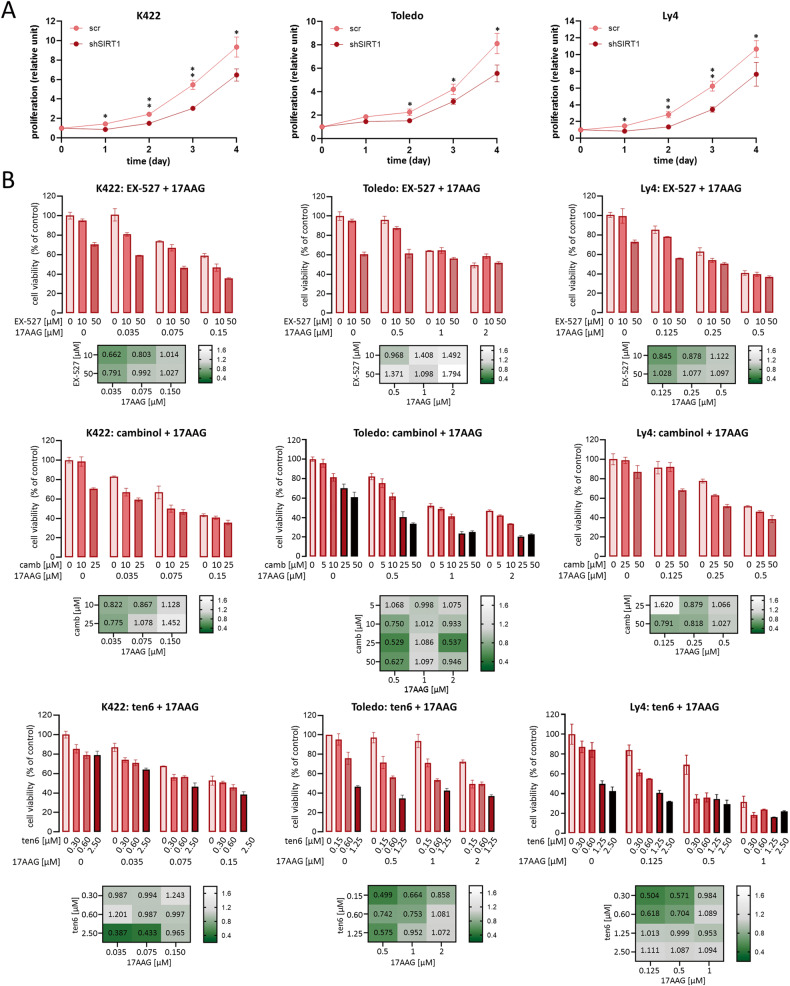
Combined SIRT1 and HSP90 inhibition causes synergistic/additive cytotoxic effects in OxPhos-DLBCL cell lines: K422, Toledo and Ly4. **A** SIRT1 knockdown with shRNA significantly decreases the proliferation of K422, Ly4 and Toledo. Cells were seeded at the concentration of 0.2 × 10^6^ cells/ml and counted for the four consecutive days using Trypan Blue exclusion. Graphs show averaged values ± SDs from a representative of three independent experiments performed in triplicates. Statistical analyses were performed using *t* test; ** for *p* < 0.01 and * for *p* < 0.05. **B** HSP90 and SIRT1 inhibitors exhibit synergistic/additive effects in OxPhos-DLBCL cell lines K422, Toledo and Ly4. Cell viability was assessed using MTS assay. Graphs show averaged data from three independent experiments ± SDs. CI - combination index. CI values between 0 and 0.9 indicate synergy, CI between 0.900 and 1.100 indicates additivity, and CI ≥ 1.100 indicates antagonism.

While genetic instability is generally thought to promote tumorigenesis, abnormal chromosome segregation during cell division promotes cell death (“mitotic catastrophe”) [39, 40]. As shown in Fig. 6A, disruption of SIRT1 expression significantly decreased the proliferation of OxPhos-DLBCL cell lines (K422, Ly4 and Toledo), irrespective of their *TP53* mutation status (Supplementary Table S5). In contrast, the knockdown of SIRT1 expression did not alter the proliferation of OxPhos-independent cell lines DHL4 and Ly1 (Supplementary Fig. S7A).

Given the feed-forward loop linking SIRT1 and HSP90α, we hypothesized that simultaneous blocking of SIRT1 and HSP90 activity would exhibit synergistic effects in targeting the OxPhos-DLBCL cells. To investigate this hypothesis, we assessed the cytotoxic effect of HSP90 and sirtuin inhibitor combinations (17AAG and EX-527, cambinol or tenovin-6). Simultaneous inhibiton of HSP90 and SIRT1 exhibited predominantly synergistic or additive activity in OxPhos-dependent cell lines, regardless of their TP53 mutation status (Fig. 6B, Supplementary Table S5). In contrast, HSP90 and sirtuin inhibitor combinations did not synergize in OxPhos-independent DLBCL cell lines (DHL4, DHL6, Ly1, Supplementary Fig. S7B).

## Discussion

In this work, we characterize a feed-forward loop linking HSP90α with SIRT1 deacetylase, sustaining high expression of both proteins. In addition, we identify a cooperative role of HSP90α and SIRT1 in safeguarding the integrity of mitotic chromosome segregation in DLBCL cells. Consistent with these findings, simultaneous genetic or chemical inhibition of these proteins synergized in increasing the number of aberrant metaphases and anaphases with multipolar spindle formation, anaphase bridges and lagging chromosomes.

HSP90α and SIRT1 are stress-regulated proteins, induced or activated by different cellular insults, including oxidative stress or chemotherapy. Both proteins are also engaged in regulation of multiple critical lymphoma oncogenic pathways, such as BCL6, MYC, TP53/DNA damage response [23, 25, 41–49]. More recently, oncogenic HSP90 was shown to foster metabolic adaptations of DLBCL cells required for biomass accumulation and mitochondrial energy production [41]. Importantly, SIRT1 also regulates mitochondrial biogenesis and OxPhos through the PGC1α/PPARγ pathway [50]. Through increased metabolic fluxes, biosynthesis and biomass accumulation, SIRT1 and HSP90 secure substrate and energetic needs for proliferating cells and are considered molecular links between metabolism and cell growth/proliferation [51, 52]. Of note, SIRT1 has been previously shown to act as a negative regulator of the centriole duplication in a mechanism involving Polo-like and Aurora A kinases in human osteosarcoma cell line [38, 54, 55]. Although the role of HSP90 in regulation of mitosis, to our knowledge, has not been raised before, previous in silico analyses of proteins chaperoned by tumor-enriched HSP90 isoforms demonstrated that HSP90 interacts with multiple proteins involved in G2/M DNA damage checkpoint regulation, cell cycle control and chromosomal replication [21]. Consistent with these findings, our studies provide experimental and functional evidence of the HSP90α role in ensuring proper chromosomal segregation in mitosis in DLBCL cells.

In certain tumors including DLBCLs, HSP90 organizes into higher order complexes termed epichaperomes that are more stable than the classical folding chaperome complexes characteristic for normal cells [18, 19, 41]. These findings highlight the role of HSP90 in maintaining cancer proteome homeostasis, in particular - of the proteins constituting aforementioned cancer hallmark - related pathways.

The feed-forward loop linking these proteins suggests that their increased levels cooperatively support tumor cell survival by regulating a subset of common processes including metabolism, transcription, translation, cell cycle, DNA damage responses and mitosis [25, 53]. Safeguarding mitosis integrity, identified in our study, extends the list of known pathways regulated by SIRT1 and HSP90 in DLBCL cells. In addition, we demonstrate their cooperative role in this process. In this aspect, SIRT1, augmenting HSP90 expression, might play an important function in assuring proper levels of HSP90 to form key chaperomes/epichaperomes in cancer cells exposed to multiple external and internal stressors, resulting from changing microenvironmental conditions or therapy.

**Fig. 7:**
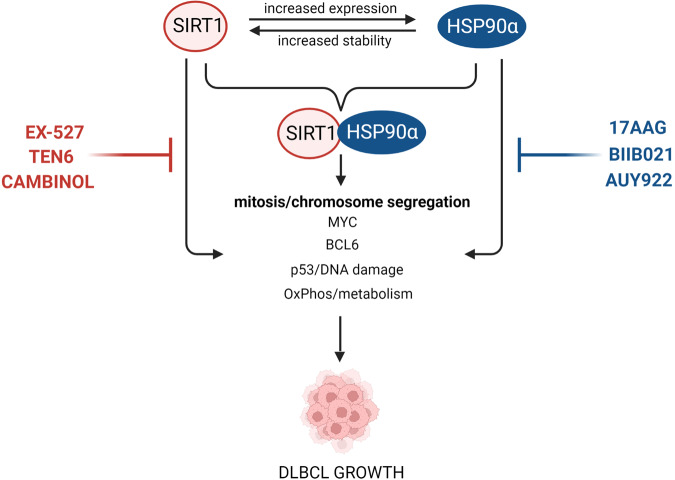
Feed-forward loop between SIRT1 and HSP90α in DLBCL cells. SIRT1 contributes to HSP90α upregulation by inducing its gene expression. In turn, HSP90α chaperones SIRT1 protein and enhances its stability. Individually or as a complex, SIRT1 and HSP90α support key oncogenic pathways in DLBCL. Created with BioRender.

Our observations indicate that targeting SIRT1 and HSP90α individually or in combination would block critical survival/oncogenic pathways and likely produce a synergistic effects (Fig. 7). Combinatorial inhibition of SIRT1 and HSP90α is also a rational approach from a mechanistic standpoint, since SIRT1 and HSP90 regulate their subordinate pathways in entirely distinct manners (i.e., either through deacetylation of substrates, or by chaperoning or fostering nucleation of multiprotein complexes, respectively). Given the variety of molecular consequences evoked by these inhibitors, inhibition of SIRT1 and oncogenic HSP90 circuit will likely collapse cellular epichaperomes and disturb the broad spectrum of their subordinate processes, decreasing cancer cell metabolic fitness, proliferation and viability. In this aspect, abnormal mitoses caused by SIRT1/HSP90 blockade are certainly important contributors to lymphoma cell death. Noteworthy, abnormal mitoses occurred in 30–50% of cells treated with chemical SIRT1/HSP90 inhibitors or with their genetic silencing over the period of only one cell division, suggesting that the accumulation of these events over prolonged exposure time might lead to a mitotic catastrophe - a cell death mechanism lacking a clear definition, but characterized by abnormalities in the mitotic apparatus, dysfunction of the mitotic checkpoint, and failure in the completion of normal mitosis [56, 57]. In line with this hypothesis, mitotic catastrophe can be evoked by depletion of centrosomal proteins, which are regulated by SIRT1 [54, 58]. Importantly, consistent with the p53-independent nature of mitotic catastrophe, the HSP90/SIRT1 inhibition-induced cell death was p53-independent.

The overexpression and mitotic consequences of the HSP90/SIRT1 circuit inhibition were more typical for COO-independent DLBCL subgroup characterized by an increased OxPhos signature and a distinct energy source utilization (OxPhos DLBCLs). These observations further underscore the role of metabolic programming of cancer cells as a prerequisite of active proliferation. Interestingly, SIRT1 and HSP90α modulate both these processes in ensemble, providing a coordinated control over metabolism and cell cycle. These finding also suggest potential therapeutic vulnerability in OxPhos tumors. In fact, although inhibitors of canonical survival pathways, such as BCR signaling, BCL2 and PI3K/AKT are being evaluated in distinct molecular subsets of DLBCL, there are currently no clinically approved targeted therapeutic strategies for OxPhos-type tumors [59–61], highlighting an unmet therapeutic need.

Although over 18 HSP90 inhibitors advanced to clinical trials demonstrating a broad potential to synergize with other drugs, including targeted small molecules and chemotherapeutics [52, 53, 62], there are no clinically available SIRT1 inhibitors, precluding proof-of-concept preclinical in vivo studies and clinical trial design based on the proposed combinatorial strategy. In this study, we used SIRT1 inhibitors that differ in selectivity. EX-527 (selisistat) exhibits the highest selectivity towards SIRT1 (over 200-fold weaker activity against SIRT2 and SIRT3) [63]. Cambinol was reported to inhibit SIRT1 and SIRT2 with comparable selectivity, while tenovin-6 blocks additionally SIRT3 [64]. However, since the effects of these inhibitors were reproduced using genetic SIRT1 depletion, our studies suggest that selective SIRT1 inhibitor would likely show sufficient activity and better tolerability than less selective compounds. Alternatively, since SIRT1 is NAD(+)-dependent enzyme, it is possible to target its activity indirectly, e.g., by blocking the rate-limiting enzyme in the NAD salvage pathway, nicotinamide phosphoribosyltransferase (NAMPT). Although this approach is not specific towards SIRT1, NAMPT inhibitors exhibit potent antitumor activity and acceptable safety profiles in preclinical models of hematologic malignancies [65, 66].

Taken together, our findings define a new pathogenetic circuit linking HSP90α with SIRT1, playing an important role in chromosome segregation integrity in a subset of DLBCL cells. Given the very high potential of HSP90/SIRT1 inhibitors for combinations with additional chemotherapeutics, these studies warrant further investigations of HSP90/SIRT1 pathway inhibitor combinations in OxPhos-dependent DLBCL models.

### Supplementary information

Supplementary Material

Original Data File

Reproducibility checklist

## Data Availability

Further information and requests for resources and reagents should be directed and will be fulfilled by the corresponding author, upon reasonable request.
