# Supplementary material for: SIRT1 and HSP90α feed-forward circuit safeguards chromosome segregation integrity in diffuse large B cell lymphomas
Source: Cell Death Dis. 2023 Oct 11;14(10):667. doi: 10.1038/s41419-023-06186-0 (PMC10564908; doi:10.1038/s41419-023-06186-0)

**Supplementary Figures and Information**

Emilia Białopiotrowicz-Data^1^, Monika Noyszewska-Kania^1^, Ewa Jabłońska^1^, Tomasz Sewastianik^1^, Dorota Komar^1^, Sonia Dębek^1^, Filip Garbicz^1^, Magdalena Wojtas^2^, Maciej Szydłowski^1^, Anna Polak^1^, Patryk Górniak^1^, Przemysław Juszczyński^1^

1 – Department of Experimental Hematology, Institute of Hematology and Transfusion Medicine, Warsaw, Poland

2 – Department of Diagnostic Hematology, Institute of Hematology and Transfusion Medicine, Warsaw, Poland

**Contact information for correspondence:** Prof. Przemyslaw Juszczynski, address: Institute of Hematology and Transfusion Medicine, Department of Experimental Hematology, Chocimska 5 Str, Warsaw, 00-791, Poland; e-mail: pjuszczynski@ihit.waw.pl, tel.: +48 22 3496 699; fax: +48 22 3496 237

**Supplemental Table S1. SIRT1 targeting sequences**

| **shRNA** | **5’→3’ sequence** |
| --- | --- |
| **SIRT1 T** | GATCCGCGGGAATCCAAAGGATAATTTTCAAGAGAAATTATCCTTTGGATTCCCGCTTTTTTG |
| **SIRT1 B** | AATTCAAAAAAGCGGGAATCCAAAGGATAATTTCTCTTGAAAATTATCCTTTGGATTCCCGCG |

**Supplemental Table S2. Primers used for RQ-PCR**

| **primer** | **5’→3’ sequence** |
| --- | --- |
| **SIRT1_F** | AGTGGGACATGCCAGAGTCCA |
| **SIRT1_R** | TCCCAAATCCAGCTCCTCCAGC |
| **SIRT2_F** | TGGAATCTCCACATCCGCAG |
| **SIRT2_R** | GATGGTTGGCTTGAACTGCC |
| **HSP90AA1_F** | CCGTCGCTATATAAGGCAGGC |
| **HSP90AA1_R** | TGGGTTTCCTCAGGCATCTTGG |
| **HSP90AB1_F** | AAGTGCACCATGGAGAGGAGG |
| **HSP90AB1_R** | GCGAATCTTGTCCAAGGCATCAG |
| **18S_F** | CGTCTGCCCTATCAACTTTG |
| **18S_R** | TGCCTTCCTTGGATGTGGTAG |

**Supplemental Table S3. Primers used for construction of vectors with SIRT1 fragments**

| **primer** | **DNA fragment** | **5’→3’ sequence** |
| --- | --- | --- |
| **N-SIRT1_F** | **N-SIRT1  (FLAG-tag on C’)** | CCCAAGCTTATGGCGGACGAGGCGGCCCT |
| **N-SIRT1_R** |  | CGCGGATCCTTACTTATCGTCGTCATCCTTGTAATCAGACACCCCAGCTCCAGTTAG |
| **N-SIRT1_F** | **N-CAT SIRT1 (FLAG-tag on C’)** | CCCAAGCTTATGGCGGACGAGGCGGCCCT |
| **CAT-SIRT1_R** |  | CGCGGATCCTTACTTATCGTCGTCATCCTTGTAATCGGCATATTCACCACCTAACCTATG |
| **C-SIRT1_F** | **C-SIRT1 (FLAG-tag on C’)** | CCCAAGCTTATGGCCAAACTTTGCTGTAACCCTGTA |
| **C-SIRT1-R** |  | CGCGGATCCTTACTTATCGTCGTCATCCTTGTAATCCTCTTGATCATCTCCATCAGTCCC |
| **HSP90AA1-HA_F** | **HSP90α-HA  (HA-tag on C’)** | CGCGGATCCATGCCTGAGGAACCCAGACCC |
| **HSP90AA1-HA-R** |  | GCTTGCGGCCGCTTAAGCGTAATCTGGAACATCGTATGGGTTGTCTAGTTCTTCCATGCGTGATGT |

**Supplemental Table S4. Antibodies used in the study**

| **antibody** | **origin** | **company** | **catalog number** |
| --- | --- | --- | --- |
| SIRT1, clone 10E4 | mouse | Merck Millipore | 04-1557 |
| SIRT1 (1F3) (for WB) | mouse | Cell Signaling Technology | 8469S |
| SIRT2 (D4S6J) | rabbit | Cell Signaling Technology | 12672S |
| K48-linkage specific polyubiquitin antibody | rabbit | Cell Signaling Technology | 4289 |
| HSP90α | rabbit | Abcam | ab2928 |
| HSP90β | rabbit | Abcam | ab2927 |
| HSF1 | rabbit | Cell Signaling Technology | 4356S |
| anti-GAPDH, clone6C5 | mouse | Merck Millipore | MAB374 |
| FLAG M2 | mouse | Sigma-Aldrich | F1804-1MG |
| HA-Tag (C29F4) | rabbit | Cell Signaling Technology | 3724S |
| pericentrin | rabbit | Abcam | ab4448 |
| normal mouse IgG (control for CoIP) | mouse | Santa Cruz Biotechnology | sc-2025 |
| normal rabbit IgG (control for CoIP) | rabbit | Santa Cruz Biotechnology | sc-2027 |
| anti-α-tubulin | mouse | Calbiochem | CP06-100UG |
| anti-rabbit IgG Fab2 Alexa Fluor 488 | goat | Cell Signaling Technology | 4412S |
| Texas Red goat anti-mouse IgG | goat | Invitrogen | T-862 |
| Anti-rabbit IgG (whole molecule)-peroxidase | goat | Sigma-Aldrich | A0545-1ML |
| Anti-mouse IgG (whole molecule)-peroxidase | rabbit | Sigma-Aldrich | A9044 |

**Supplemental Table S5. TP53 status of OxPhos DLBCL cell lines used in the study estimated by Sanger sequencing**

| **cell line** | **TP53 status** | **exon number** | **cDNA description** | **protein change** | **effect** |
| --- | --- | --- | --- | --- | --- |
| **K422** | mutated | 9-exon | c.955A>T | p.K319* | nonsense |
| **Toledo** | mutated | 6-exon | c.592del | p.E198fs | frame shift |
| **Ly4** | wild type | - | - | - | - |

**Supplemental Figure S1. Increased expression of the HSP90AA1 gene and SIRT1 protein in OxPhos-dependent, compared to OxPhos-independent (BCR-subtype) DLBCLs. (A)** Relative HSP90AA1 transcript abundance in primary tumor samples of OxPhos-dependent (OxPhos-dep) and OxPhos-independent (OxPhos-indep) patients derived from publicly available dataset (Monti et al., 2015). Each column represents a sample and each row refers to a gene probe; columns are ordered by tumor type (OxPhos-dep and OxPhos-indep) as indicated. Color scale at the bottom indicates relative expression and standard deviations from the mean; n - number of patients. Lower: box plots illustrating differences in the HSP90AA1 gene expression between OxPhos-dependent and OxPhos-independent primary samples; the median expression is indicated by the horizontal line, bars denote ± 25-75 percentile and whiskers indicate the range. Statistical analysis was performed using the Mann-Whitney test. **(B)** Upper: Representative western blots depicting HSP90α and SIRT1 protein levels in OxPhos-dep and OxPhos-indep DLBCL cell lines. Lower: Summary graph presenting relative quantifications of band intensities from digital images. A ratio of HSP90α or SIRT1 band intensity to GAPDH band intensity for each cell line was calculated, then the individual values were normalized to the averaged value obtained from the eight cell lines, which was assigned as arbitrary value 1. Outlier Pfeiffer and Ly7 cell lines were marked with lighter colours. **(C)** Transcript abundance for HSP90α (left) and SIRT1 (right) in OxPhos- dependent and -independent DLBCL cell lines. Relative abundance of the transcripts was determined using 2^−ΔΔCT^ method, with *18S* ribosomal RNA used as a reference gene. The individual values were normalized to the average obtained from the eight cell lines, which was assigned as value 1. Outlier Pfeiffer and Ly7 cell lines were marked with lighter colour. In B and C graph bars represent averages from 3 independent experiments ± standard deviation (SD).


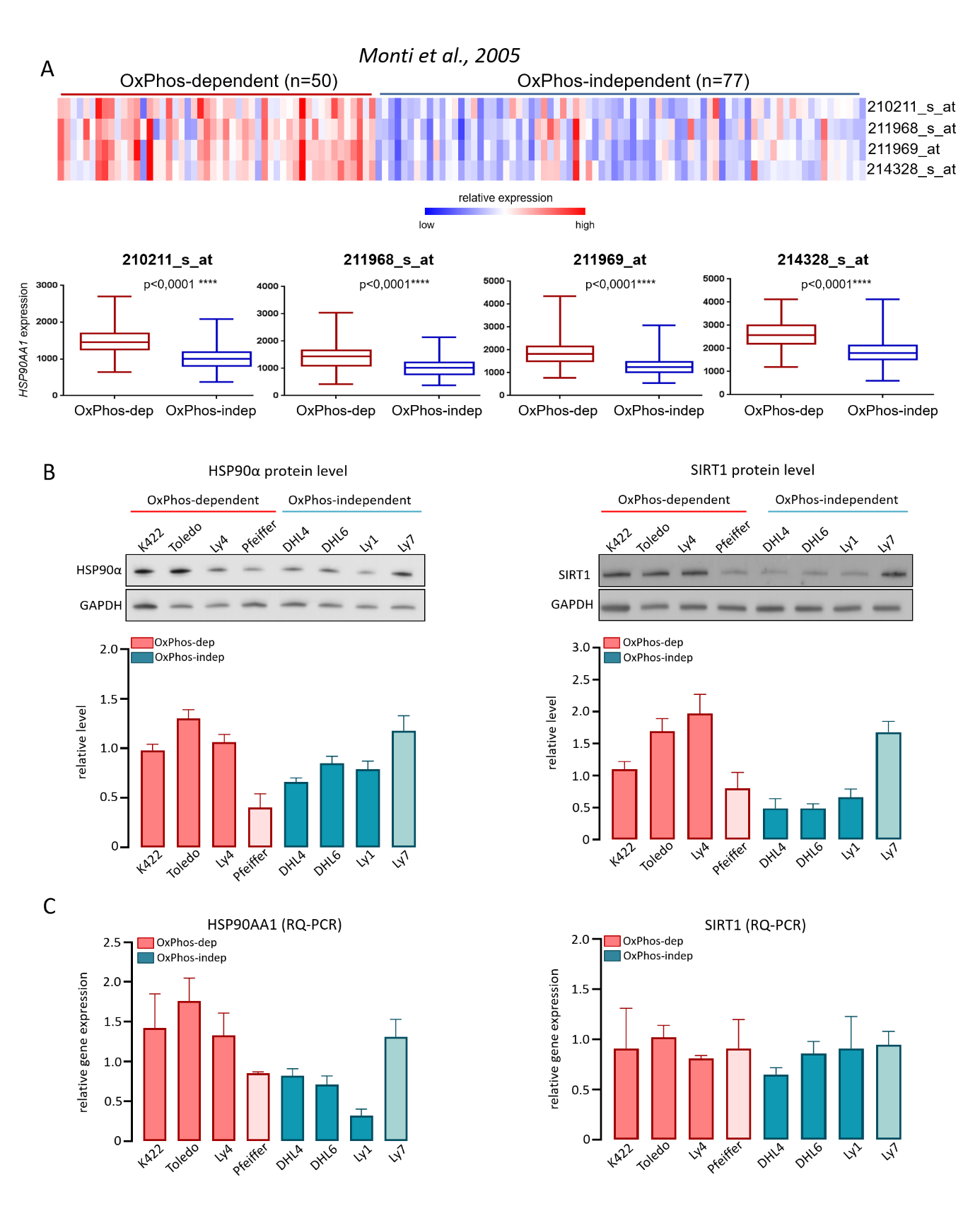


**Supplemental Figure S2. The transcript and protein levels for HSP90β cytosolic isoform do not correlate with the SIRT1 protein level in DLBCL cell lines. (A)** Upper: Transcript abundance for the HSP90β gene (HSP90AB1) in OxPhos -dependent (OxPhos-dep) and -independent (OxPhos-indep) DLBCL cell lines. Relative abundance of HSP90β transcript was determined using 2^−ΔCT^ method, with 18S ribosomal RNA used as a reference gene. The individual values were normalized to the average obtained from the eight cell lines, which was assigned as value 1. Bars indicate means ± standard deviations from at least 3 independent experiments. Outlier Pfeiffer and Ly7 cell lines were marked with lighter colours. Lower: The protein level of HSP90β is similar in OxPhos -dep and -indep DLBCL cell lines. GAPDH was used as a loading control. **(B)** Neither the HSP90β transcript (upper) nor the protein (lower) abundance correlate with the SIRT1 protein level. SIRT1 protein level was measured as a ratio of SIRT1 band intensity to GAPDH band intensity for each cell line was, then the individual values were normalized to the average obtained from the eight cell lines, which was assigned as arbitrary value 1; p - significance value, r - the Spearman correlation coefficient.


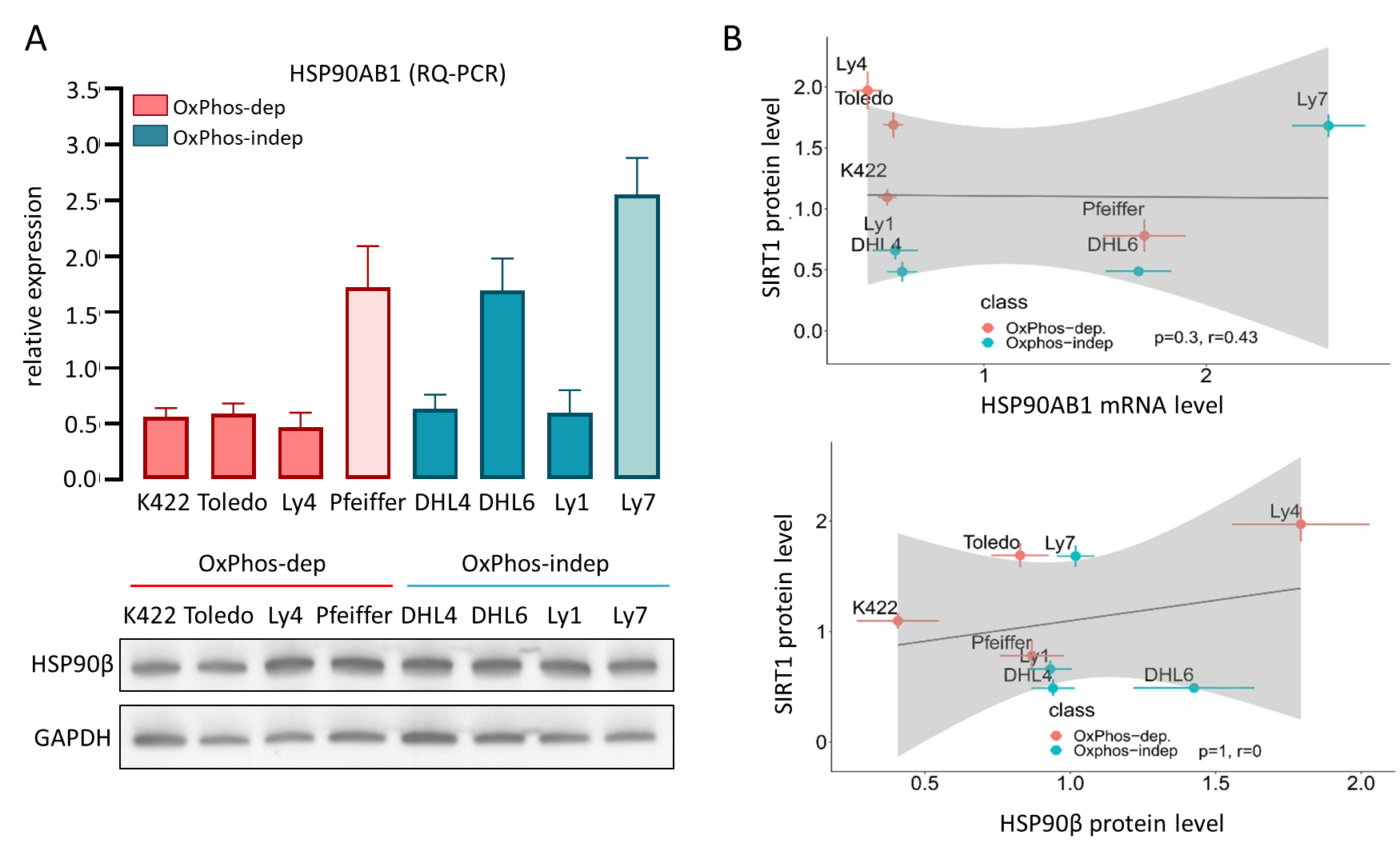


**Supplemental Figure S3.** Degradation of SIRT1 protein in time in OxPhos-dependent and OxPhos-independent DLBCL cell lines. Left: Representative western blots showing SIRT1 protein decay in time in OxPhos -dependent and -independent DLBCL cell lines. SIRT1 levels were normalized to GAPDH (loading control). Values below the blots show the results of densitometry analysis and were calculated as the % of remaining SIRT1 protein comparing to the value for time “0”, which was assigned as 100%. Right: Summary graph presenting SIRT1 protein decay in time for individual DLBCL cell lines. Averages from 3 independent experiments ± SD were shown.


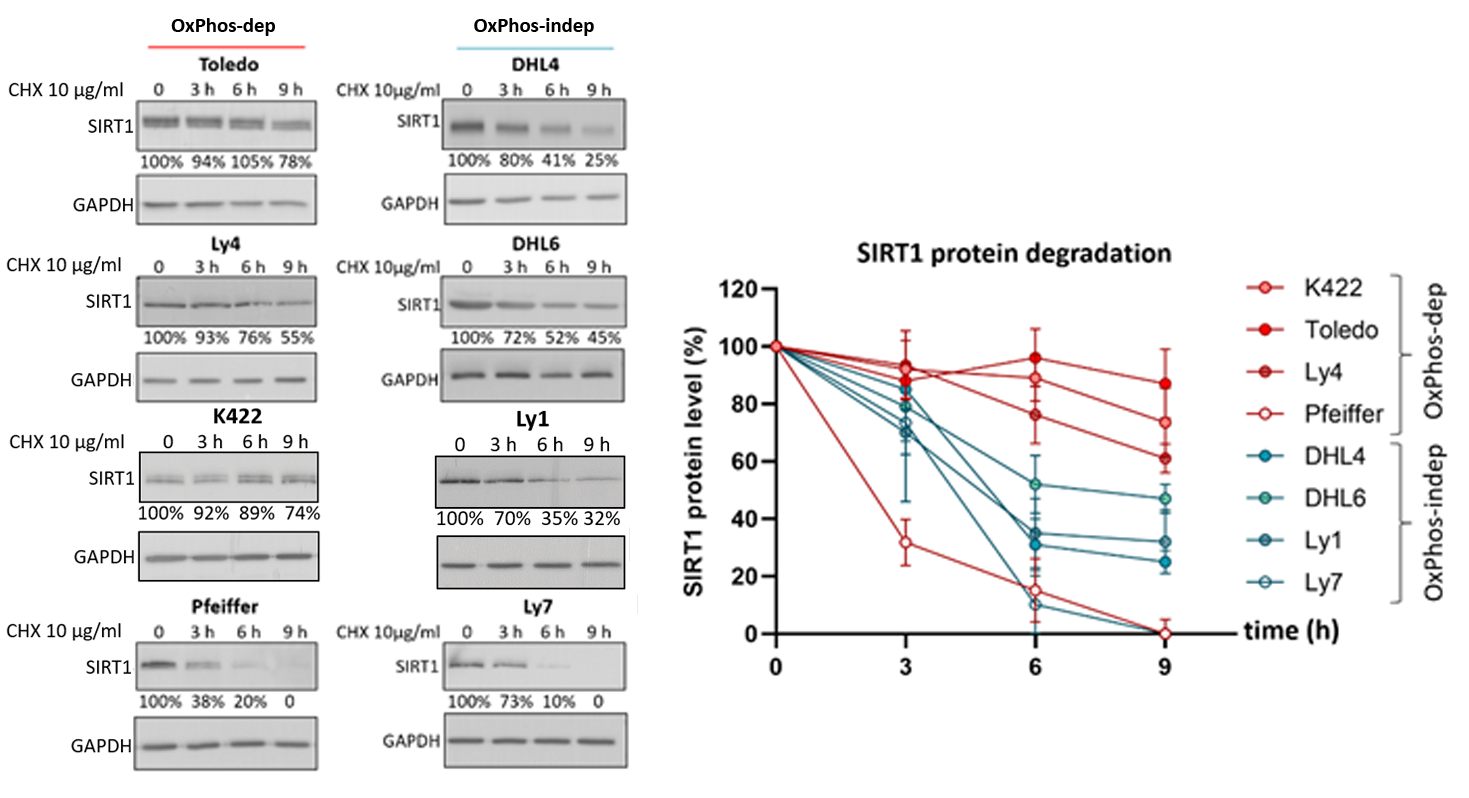
­

**Supplemental Figure S4. (A)** Negative control indicating the specificity of PLA probes used for analyses of SIRT1-HSP90α interaction. Fixed and permeabilized HEK293T cells overexpressing SIRT1-FLAG and HSP90α-HA were used in PLA experiment without the incubation with anti-SIRT1 and anti-HSP90α primary antibodies. No PLA dots are present; representative mage, 50 x magnification. The nuclei were stained in blue (DAPI) and the actin filaments were in green (Alexa Fluor 488 phalloidin). **(B)** The number of PLA dots referring to SIRT1-HSP90α complexes per cell for individual DLBCL cell lines. The results from a representative of three independent experiments were shown. Each dot indicates a value for a single cell. Thirty cells were analyzed for each cell line in a single experiment.


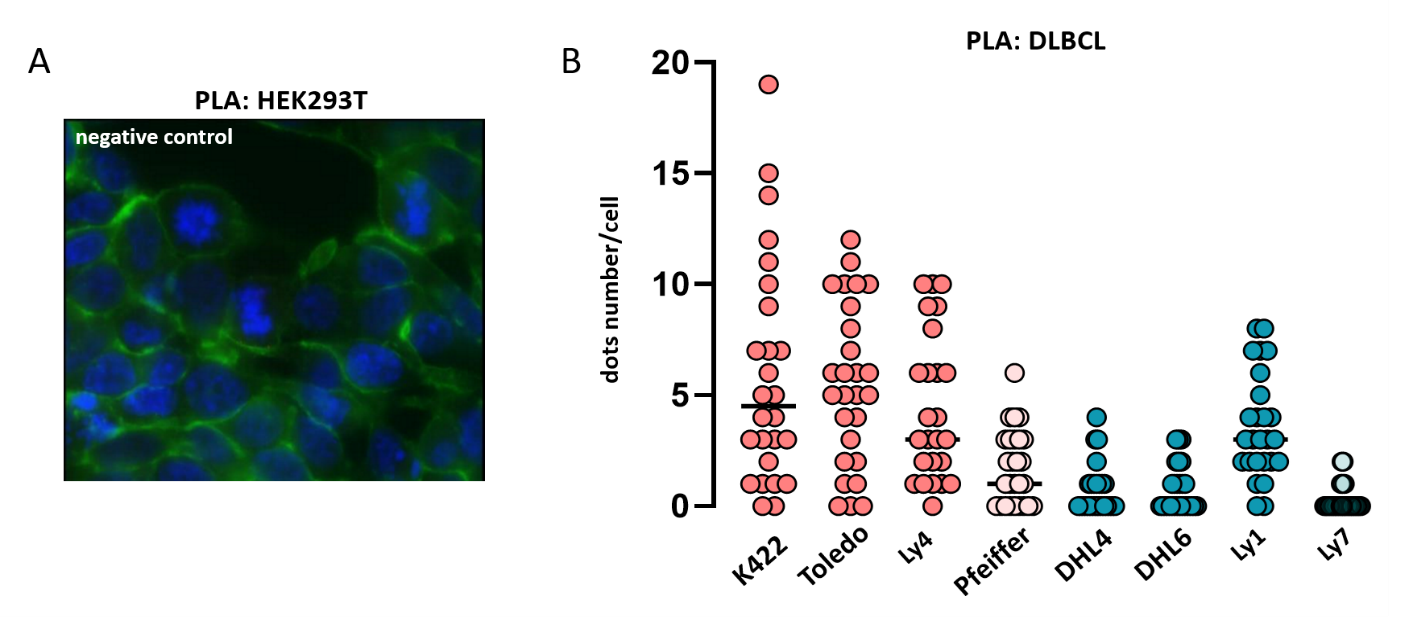


**Supplemental Figure S5. (A)** Representative images showing SIRT1-HSP90α complexes in mitotic OxPhos-independent cell line (Ly1). Mitotic cell (in metaphase) is indicated with a white arrow. **(B)** Comparison of SIRT1-HSP90α interaction in interphase and mitotic DLBCL cell lines. Graphs represent the results of one of three independent experiments. For each condition, 30 cells were analyzed. **(C)** Summary plot showing significant increase in the number of SIRT1-HSP90α complexes in mitosis versus interphase OxPhos-dependent (K422, Toledo, Ly4) cells, but not in OxPhos-independent (DHL4, DHL6, Ly1) cell lines. Each dot represents an average value from 3 cell lines ± SD. In B and C statistics was performed using paired t-test; *** for p<0.001, ** for p<0.01 and * for p<0.05.

**
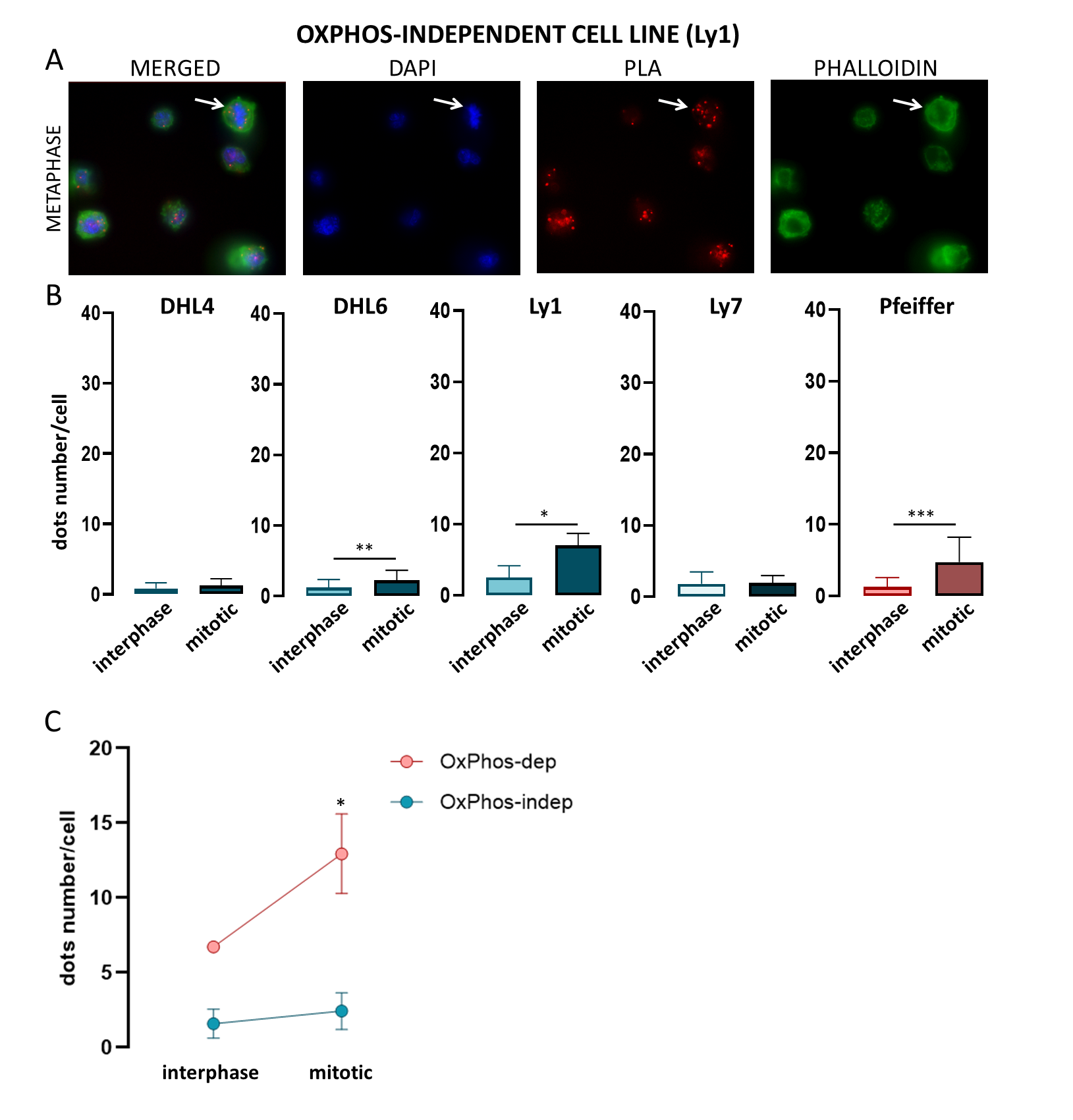
**

**Supplemental Figure S6.** Efficacy of SIRT1 and HSP90α knockdown using small interference RNA (siRNA). GAPDH was used as a loading control.

**
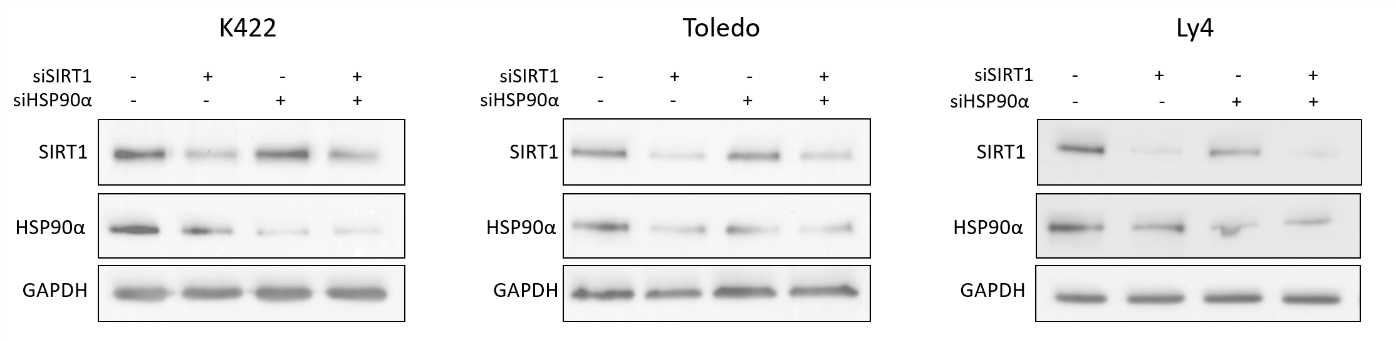
**

**Supplemental Figure S7. (A)** SIRT1 silencing does not affect the proliferation of OxPhos-independent DLBCL cell lines. The experiment was performed for OxPhos-independent DLBCL cell lines with the most efficient shRNA silencing of SIRT1 gene expression, DHL4 and Ly1. The values below the blots indicate the efficacy of SIRT1 silencing. SIRT1 protein levels were normalized to GAPDH (loading control) and referred to the control (cells transduced with scrambled shRNA), which was assigned the value 100%. For proliferation analysis, cells were seeded at a density 0.2 x 10^6^ cells/ml and live cells were counted for the next 4 days using Trypan Blue exclusion. The number of live cells in the given day was referred to the initial number of cells, which was assigned the arbitrary value 1. **(B)** The effect of combination of HSP90 inhibitor 17AAG and SIRT1 inhibitor EX-527 on the viability of OxPhos-independent: DHL4, DHL6, Ly1, and Ly7 and Pfeiffer DLBCL cell lines. Representatives of 3 independent experiments were shown. Bars exhibit means ± SDs. CI - combination index. CI values between 0 and 0.9 indicate synergy, CI between 0.900 and 1.100 indicates additivity, and CI ≥ 1.100 indicates antagonism.


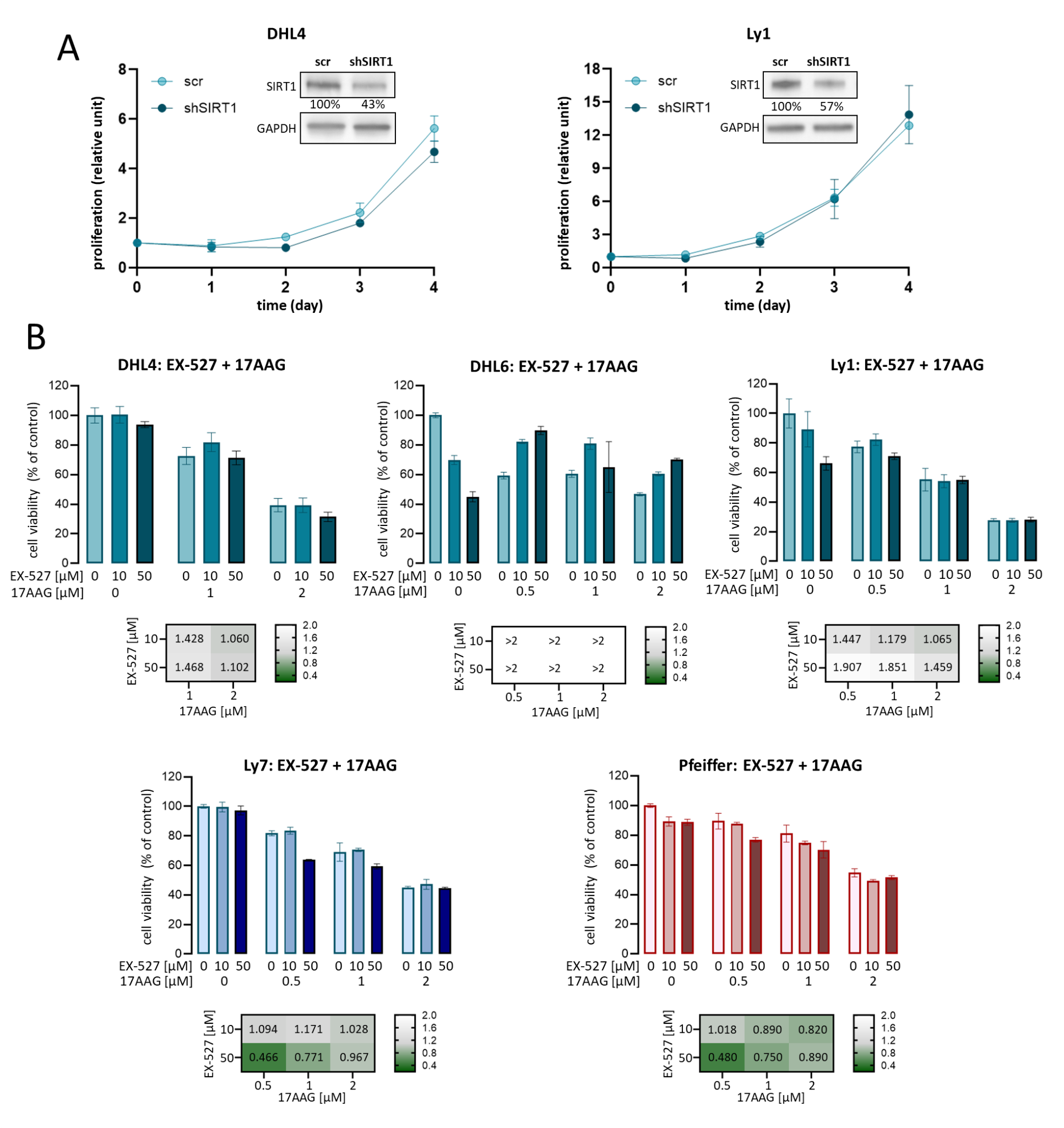

Supplement: Supplementary file 1 — Supplementary Material [file 41419_2023_6186_MOESM1_ESM.docx]
