# Supplementary material for: SIRT1 and HSP90α feed-forward circuit safeguards chromosome segregation integrity in diffuse large B cell lymphomas
Source: Cell Death Dis. 2023 Oct 11;14(10):667. doi: 10.1038/s41419-023-06186-0 (PMC10564908; doi:10.1038/s41419-023-06186-0)

Uncropped western blot images with molecular weight markers. The red dotted rectangle indicates the area shown in the corresponding manuscript Figures and Supplemental Figures.

**Figure 1A**


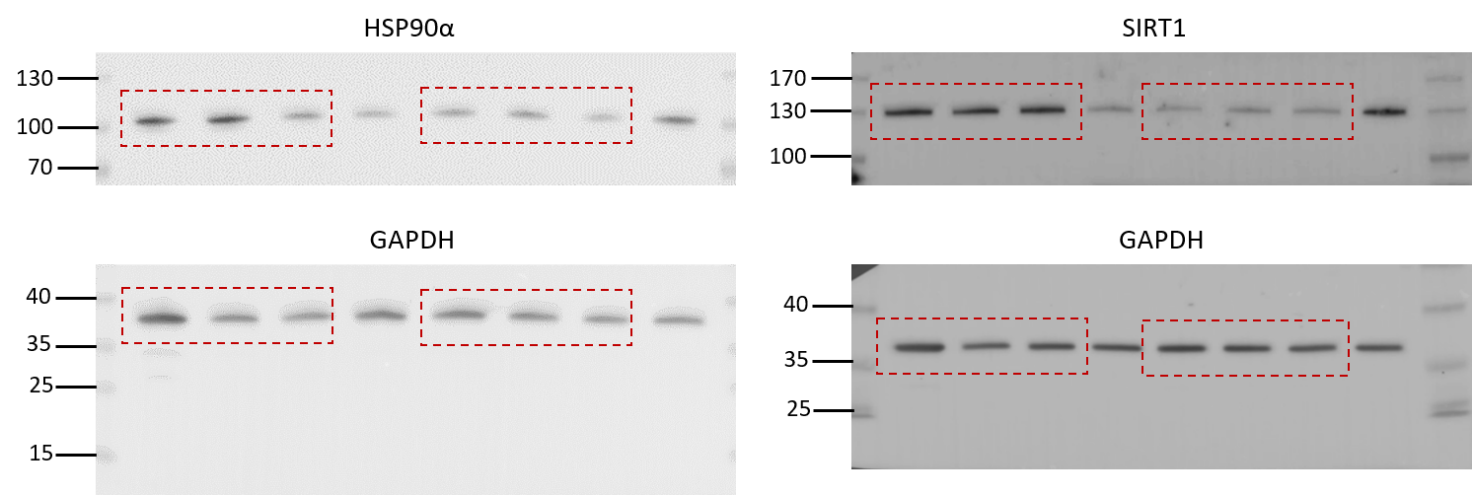


**Figure 2A**


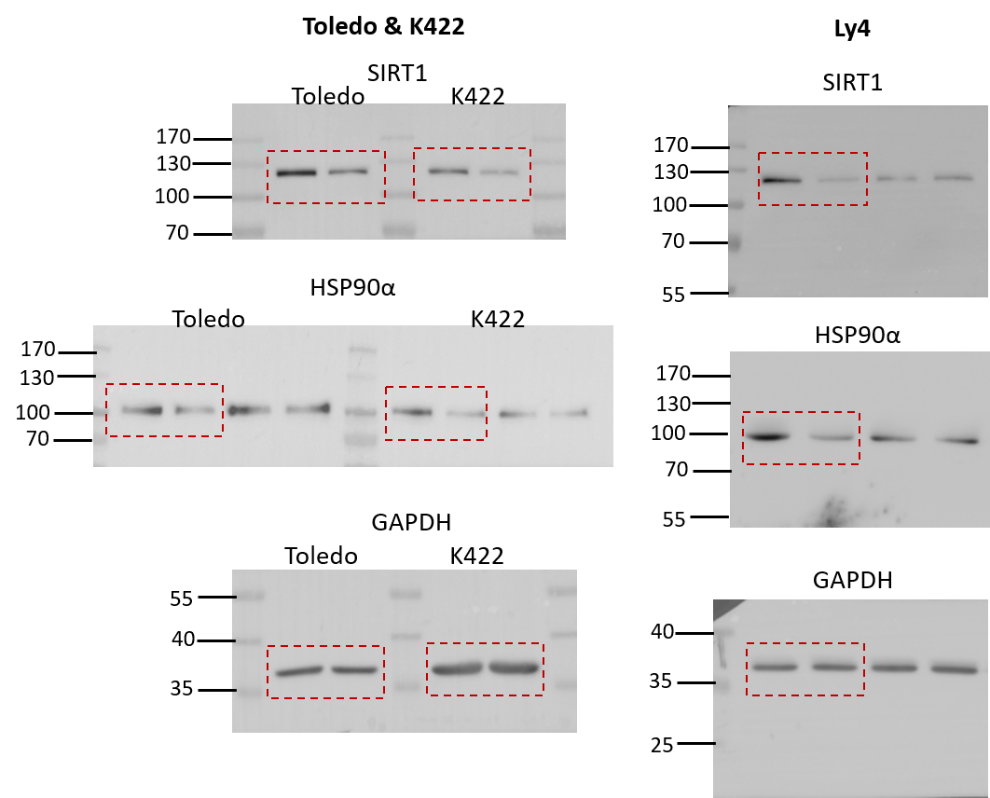


**Figure 2B**


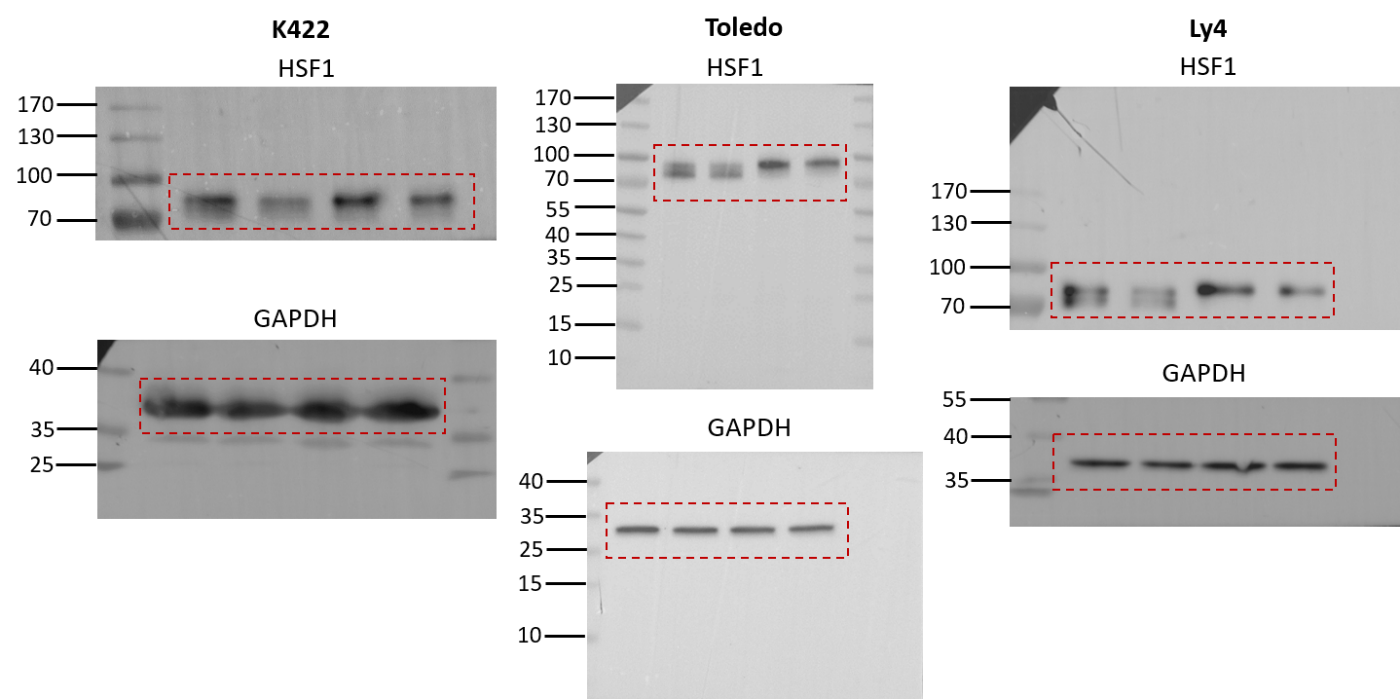


**Figure 2C**


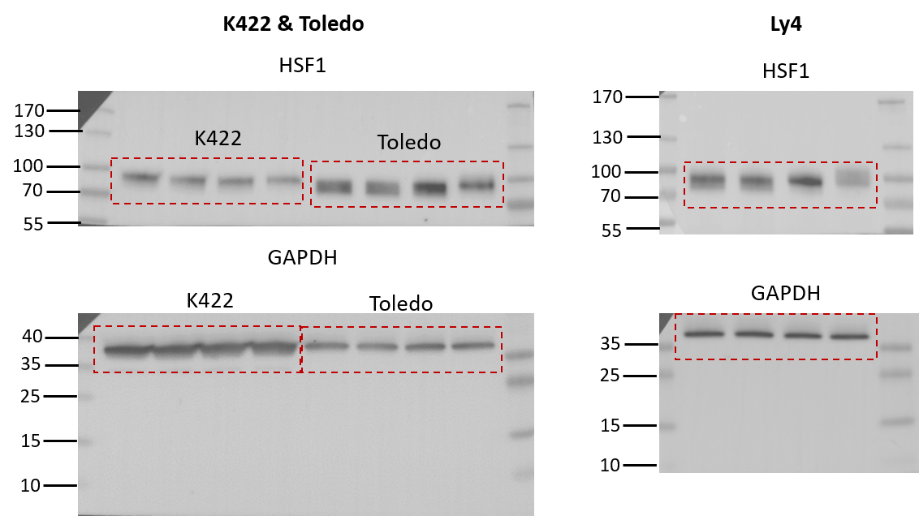


**Figure 2D**

**
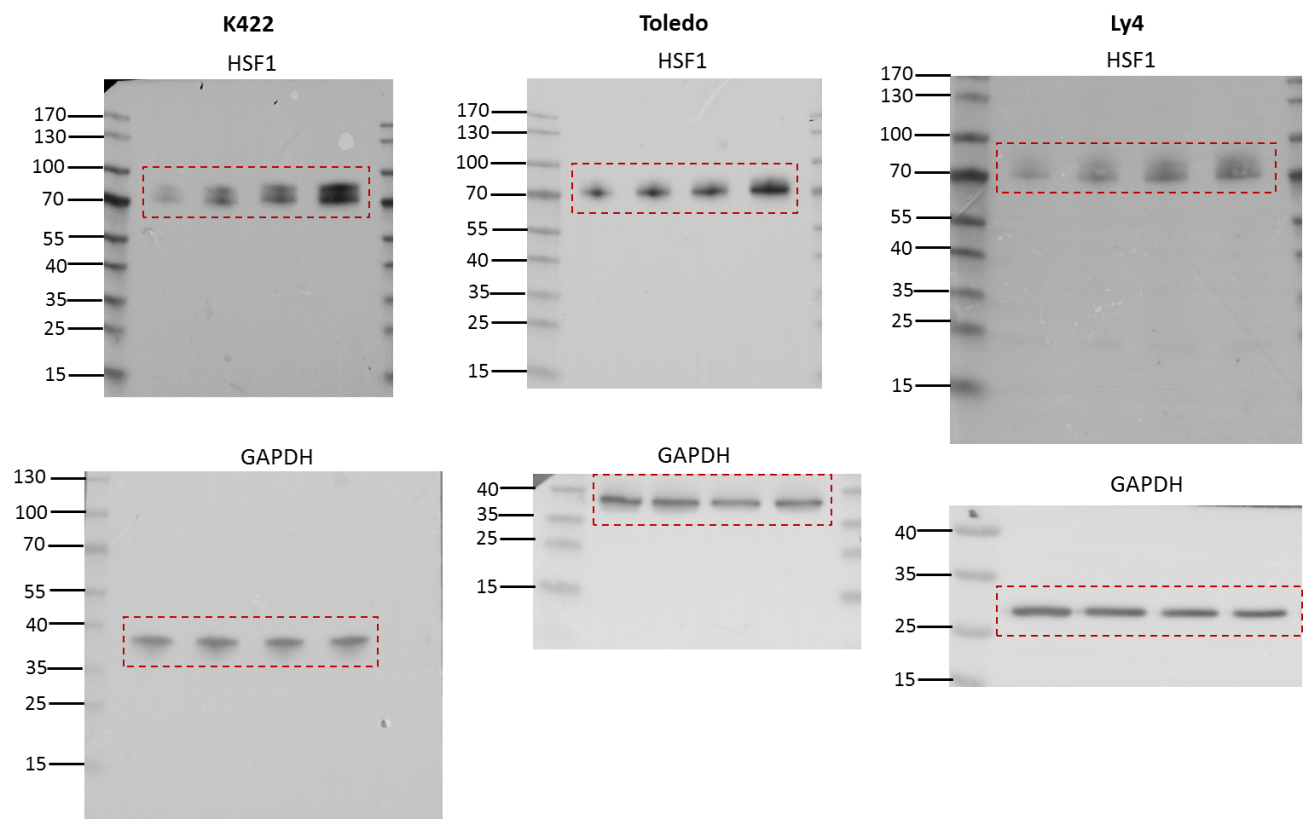
**

**Figure 3A**


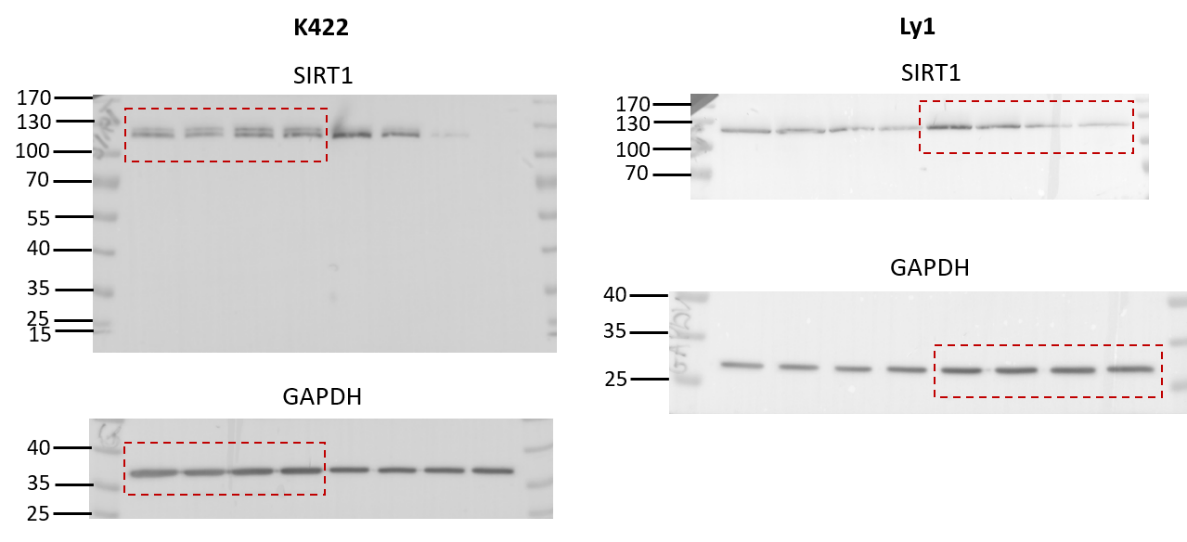


**Figure 3B**


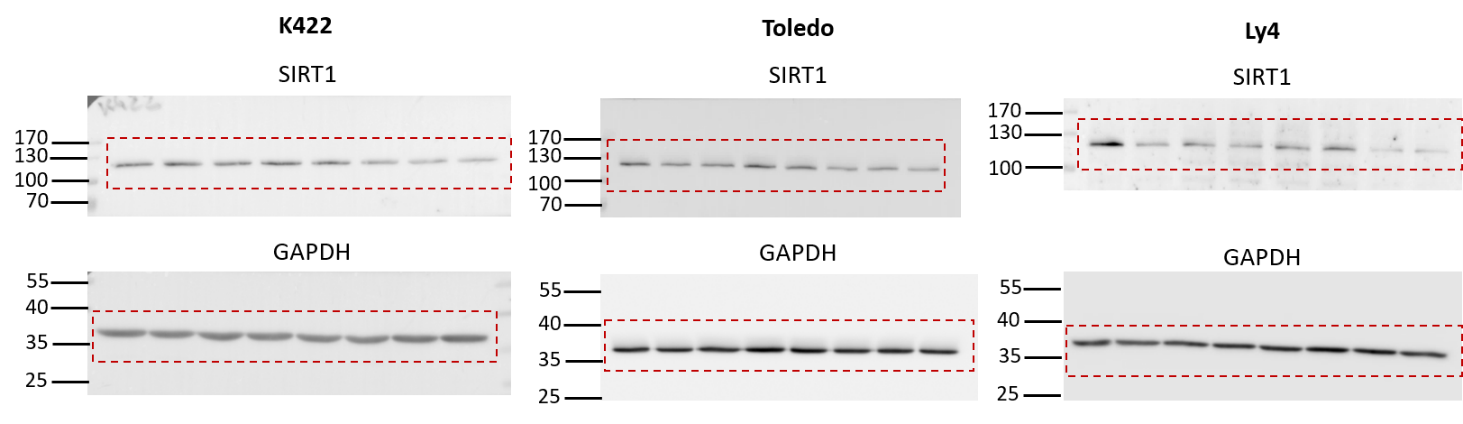


**Figure 3C**


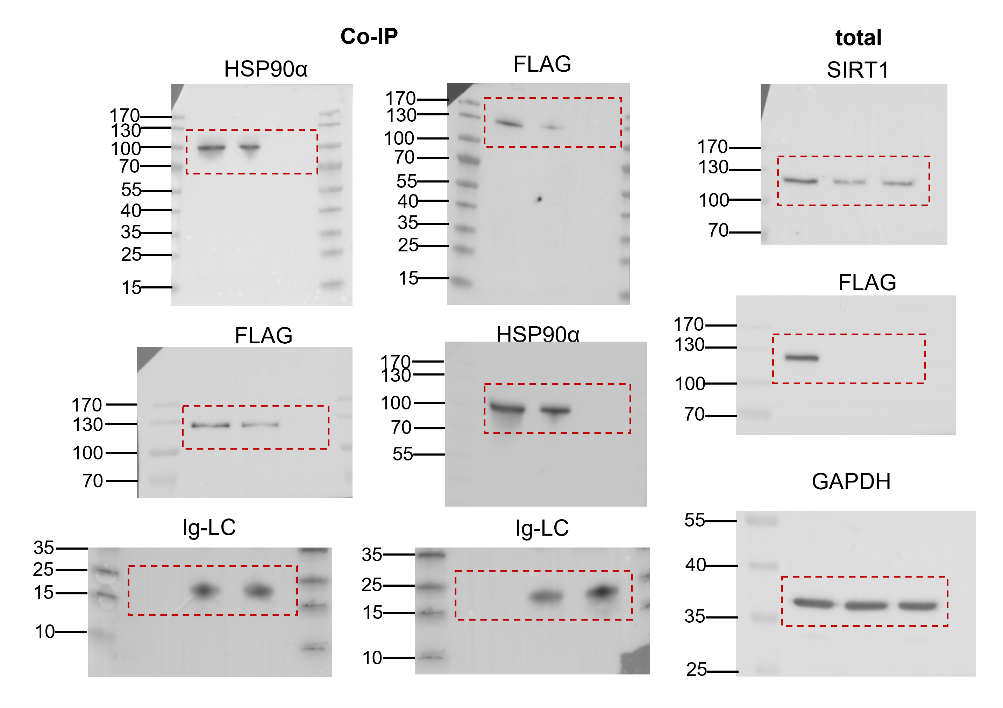


**Figure 3D**


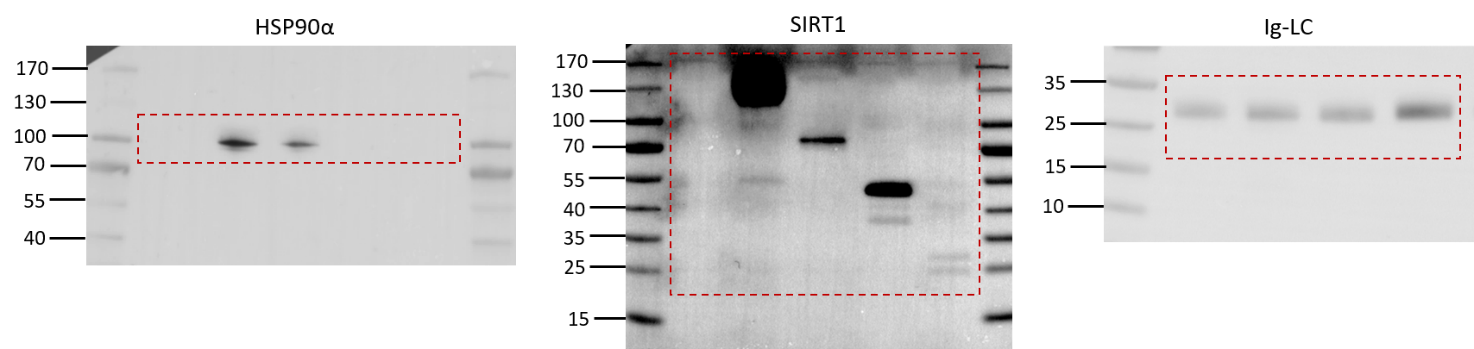


**Supplemental Figure S1**

**
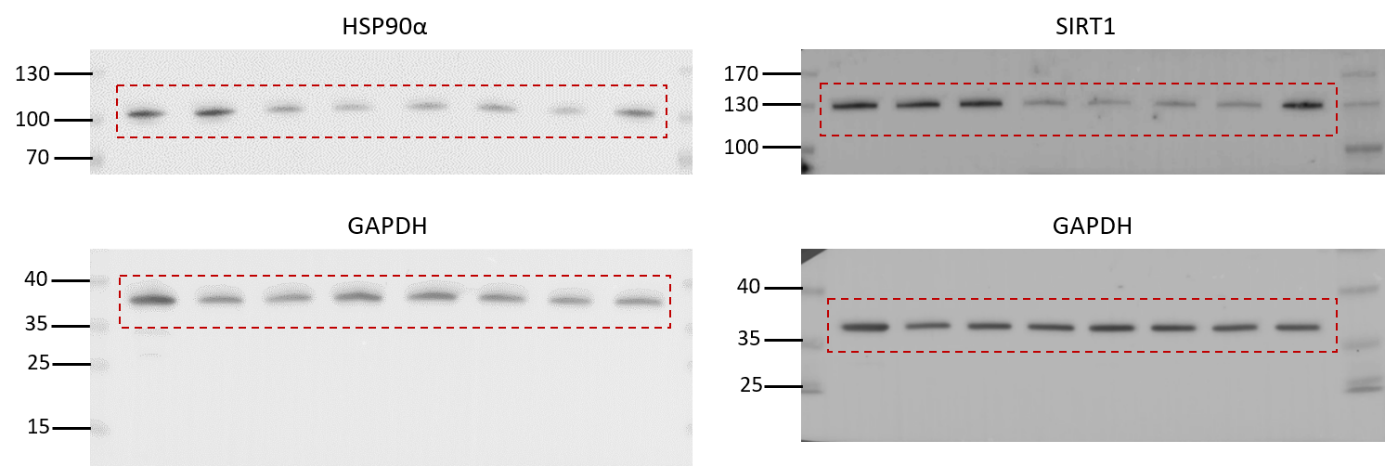
**

**Supplemental Figure S2**


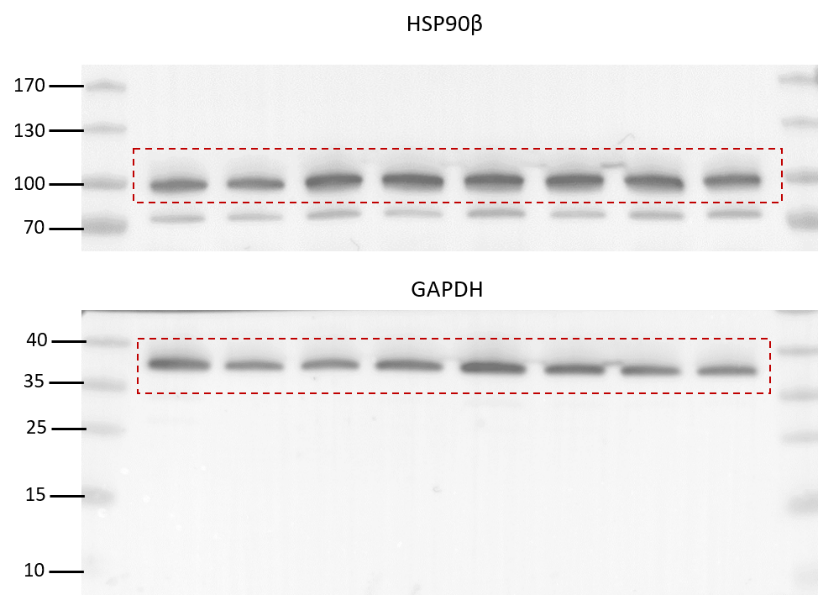


**Supplemental Figure S3**

**
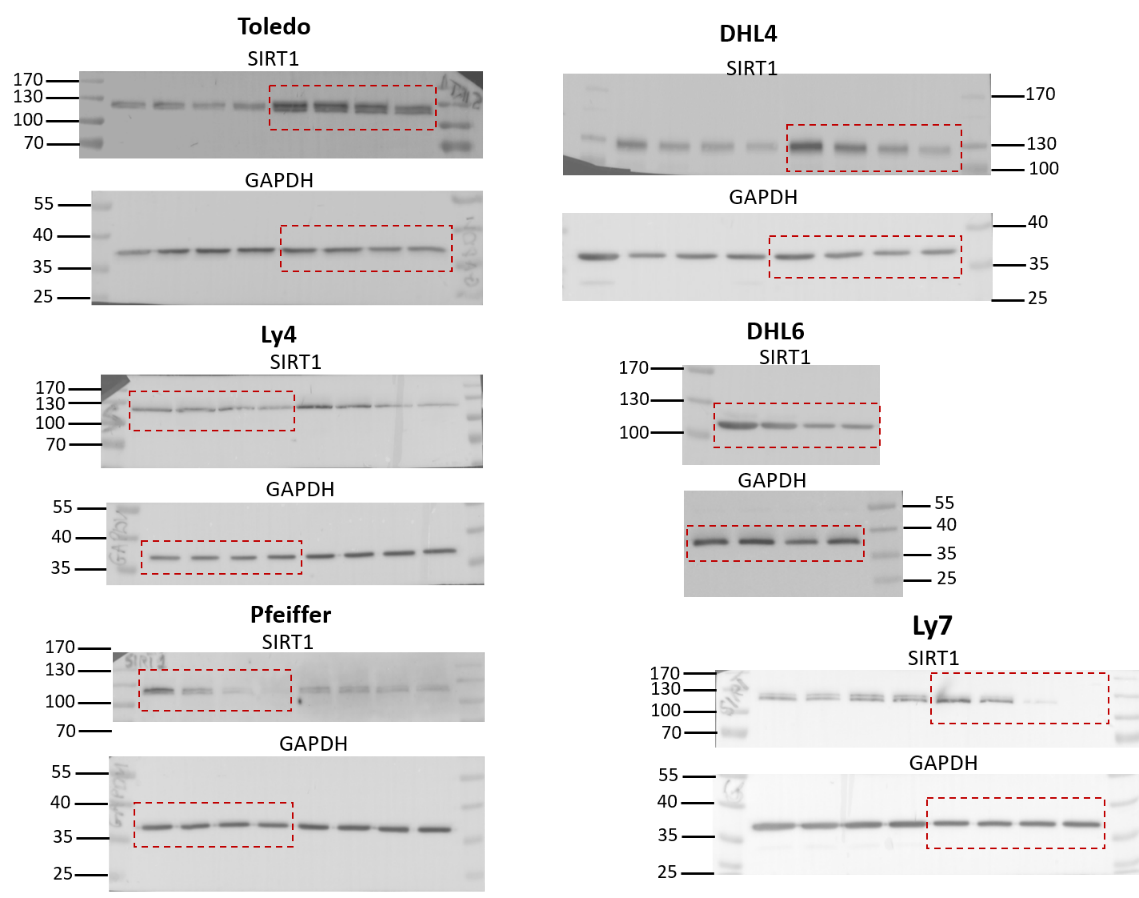
**

**Supplemental Figure S6**


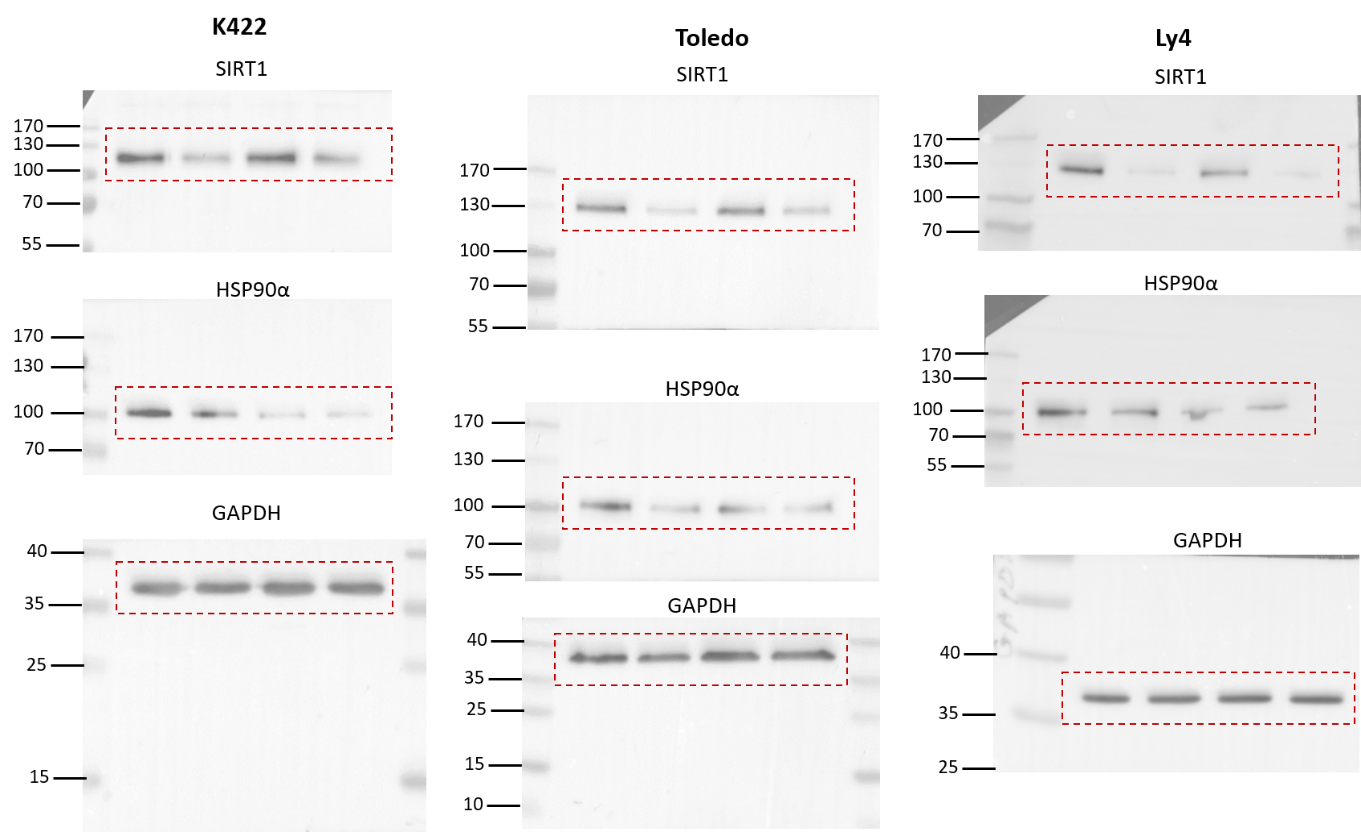


**Supplemental Figure S7A**


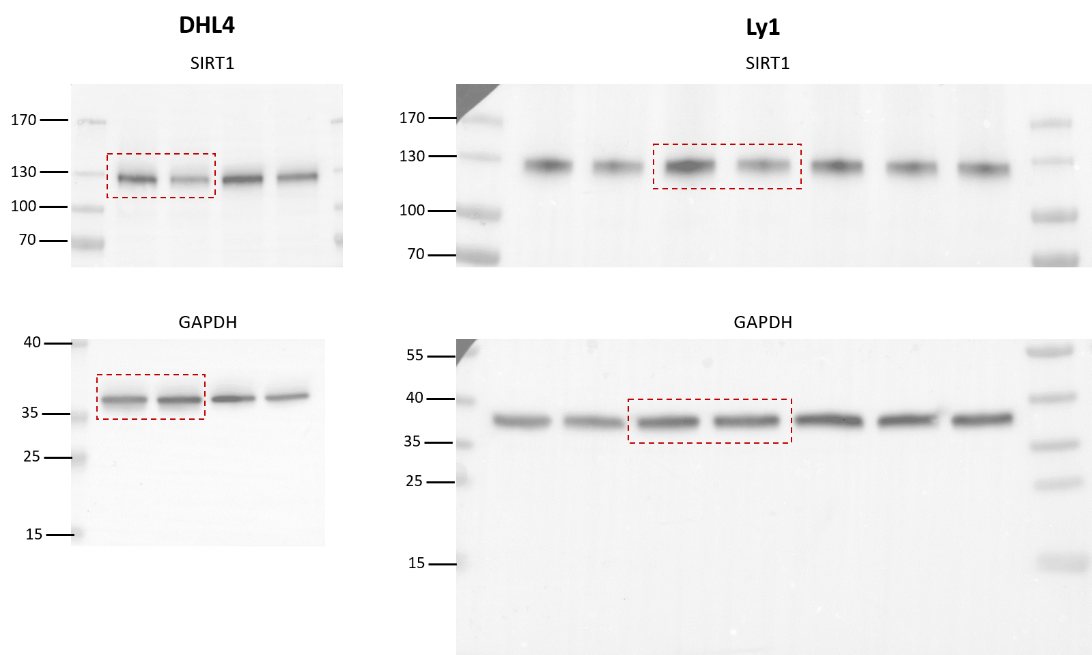

Supplement: Supplementary file 2 — Original Data File [file 41419_2023_6186_MOESM2_ESM.docx]
